# HPV-EM: an accurate HPV detection and genotyping EM algorithm

**DOI:** 10.1038/s41598-020-71300-7

**Published:** 2020-08-31

**Authors:** Matthew J. Inkman, Kay Jayachandran, Thomas M. Ellis, Fiona Ruiz, Michael D. McLellan, Christopher A. Miller, Yufeng Wu, Akinyemi I. Ojesina, Julie K. Schwarz, Jin Zhang

**Affiliations:** 1 Department of Radiation Oncology, Washington University School of Medicine, St. Louis, MO 63108 USA; 2 Department of Computer Science, Washington University in St. Louis, St. Louis, MO 63105 USA; 3 McDonnell Genome Institute, Washington University School of Medicine, St. Louis, MO 63108 USA; 4 Computer Science and Engineering Department, University of Connecticut, Storrs, CT 06269 USA; 5 Department of Epidemiology, University of Alabama at Birmingham, Birmingham, AL 35294 USA; 6 O’Neal Comprehensive Cancer Center, University of Alabama at Birmingham, Birmingham, AL 35294 USA; 7 Hudson-Alpha Institute for Biotechnology, Huntsville, AL 35806 USA; 8 Siteman Cancer Center, Washington University School of Medicine, St. Louis, MO 63108 USA; 9 Department of Cell Biology and Physiology, Washington University School of Medicine, St. Louis, MO 63108 USA; 10 Institute for Informatics, Washington University School of Medicine, St. Louis, MO 63108 USA

**Keywords:** Computational biology and bioinformatics, Prognostic markers

## Abstract

Accurate HPV genotyping is crucial in facilitating epidemiology studies, vaccine trials, and HPV-related cancer research. Contemporary HPV genotyping assays only detect < 25% of all known HPV genotypes and are not accurate for low-risk or mixed HPV genotypes. Current genomic HPV genotyping algorithms use a simple read-alignment and filtering strategy that has difficulty handling repeats and homology sequences. Therefore, we have developed an optimized expectation–maximization algorithm, designated HPV-EM, to address the ambiguities caused by repetitive sequencing reads. HPV-EM achieved 97–100% accuracy when benchmarked using cell line data and TCGA cervical cancer data. We also validated HPV-EM using DNA tiling data on an institutional cervical cancer cohort (96.5% accuracy). Using HPV-EM, we demonstrated HPV genotypic differences in recurrence and patient outcomes in cervical and head and neck cancers.

## Introduction

Human papillomavirus (HPV) infection is causative for ~ 630,000 new cancer cases globally each year^1^. Of these, HPV-associated cervical cancer is the most common primary site, with ~ 500,000 new cervical cancer diagnoses and ~ 300,000 cervical cancer-associated deaths worldwide annually^1–3^. Among head and neck cancers, a growing portion of cases are also attributed to HPV in the United States and other more developed countries^1^. In addition, HPV accounts for a substantial fraction of cancers at other anogenital sites. Currently, > 200 HPV genotypes have been identified, and ~ 15 high-risk HPV genotypes are well-established as causative agents in cancers^4–6^. Highly sensitive and accurate HPV detection and genotyping are crucial in facilitating epidemiology studies, vaccine designs, and clinical trials^7^.

Existing HPV genotyping methods are limited in the capacity to accurately identify HPV genotypes, particularly for rare genotypes. The National Cancer Insititute (NCI) recently developed a new HPV genotyping assay for detecting 51 HPV genotypes^8^. Benchmarking using the Roche Linear Array that detects 37 HPV genotypes, the NCI assay achieved a high agreement rate (~ 95%) for samples with a single high-risk HPV genotype, but the agreement rates for samples with low-risk HPV genotypes and mixed HPV genotypes varied (~ 40–90%). Multiple factors could affect the accuracy of array-based HPV detection and genotyping methods, including variability in viral load, consensus primer homology, unbalanced primer sharing, and differential amplification efficiencies^8^. The National Institute of Allergy and Infectious Diseases (NIAID) has recently developed a papillomavirus genome database (PaVE)^5, 6^, which has facilitated the creation of HPV genotyping tools using non-array-selected genomic sequencing data^9, 10^. However, genomic methods that adopt a simple read-alignment and filtering strategy can easily encounter difficulties in handling repeats and homology sequences. Recently, an accurate HPV genotyping algorithm was developed using metagenomic DNA data, which uses a strategy of masking simple repeats and homology sequences^10^; however, this methodology does not take into account expression of HPV related proteins, including the capacity of some viral strains to express alternatively spliced isoforms of key oncogenic proteins^11, 12^. To address these shortcomings of currently available HPV genotyping methods, we have focused our efforts on developing an accurate HPV detection and genotyping algorithm using whole transcriptome sequencing (RNA-seq) data.

To specifically address the ambiguities caused by repeats and homology^10^, we designed a novel expectation–maximization (EM) algorithm, designated HPV-EM, to calculate an optimized mapping of RNA-seq reads to HPV reference genomes based on a probability model. Genotyping using RNA-seq data has an advantage over using DNA data, as it enables us to simultaneously measure HPV gene expression. While the read masking strategy in the previous method^10^ would lose data and so is not suitable for estimating HPV gene expression, our HPV-EM algorithm uses all the available data and accurately estimates HPV gene expression. HPV-EM is able to evaluate HPV gene expression in samples with either single or mixed HPV genotypes based on its accurate genotyping prediction. To facilitate functional analysis for HPV induced cancers, we also implemented an automated visualization tool to show the read coverage for each HPV genotype within a sample. We benchmarked HPV-EM using cervical cancer cell line data, simulated data, and TCGA-CESC data, and validated HPV-EM using an institutional cohort of cervical cancer samples (with both RNA-seq data and DNA tiling array data). To demonstrate its broader applicability, we also applied HPV-EM to TCGA-HNSC cohort. Using this novel tool, we demonstrate HPV genotype-specific differences in recurrence and patient outcomes in both cervical and head and neck cancers. Here, we present the details of the design and implementation our accurate EM algorithm and the benchmarking experiments and applications. The HPV-EM tool is free and open source software and can be downloaded at: https://github.com/jin-wash-u/HPV-EM.

## Material and methods

### Overview of the HPV-EM tool

**Figure 1:**
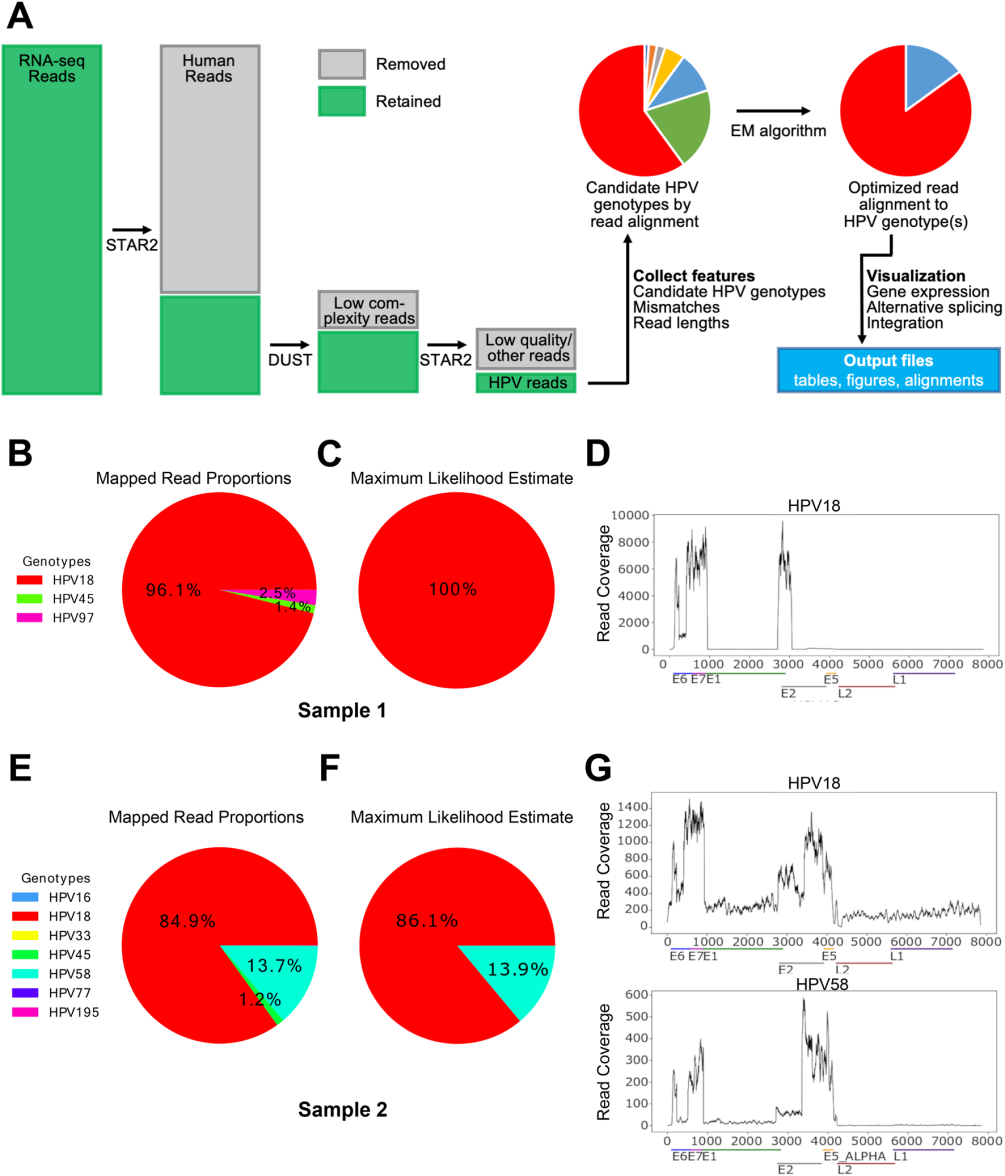
Overview and use cases of the HPV-EM tool. (**A**) Overview of the HPV-EM tool. (**B**) Proportions of aligned HPV reads of Sample 1. (**C**) HPV-EM estimated HPV genotypes in sample 1. (**D**) HPV read coverage for sample 1. (**E**) Proportions of aligned HPV reads of Sample 2. (**F**) HPV-EM estimated HPV genotypes in sample 2. (**G**) HPV read coverage for sample 2. Samples 1 and 2 are from the TCGA cervical cancer cohort. B–D show the scenario of one HPV genotype. Reads are aligned to multiple genotypes, but after HPV-EM’s optimization, 100% of HPV reads are assigned to the single correct HPV genotype. (**E**–**G)** show the scenario of mixed HPV genotypes. After HPV-EM’s optimization, HPV reads are assigned to the two correct HPV genotypes. In B and E, the keys show all genotypes with aligned HPV reads before using HPV-EM. Those with very low frequencies are not visible in the pie charts. (**D**,**G**) show HPV-EM’s visualization of HPV genes and read coverage per nucleotide.

The HPV-EM tool is designed to detect HPV gene expression and estimate the makeup of HPV genotypes present in a sample that is sequenced using whole transcriptome sequencing (RNA-seq). The input of HPV-EM includes RNA-seq reads in FASTQ format (which may contain both human and HPV sequences) and the path to a directory containing an indexed human reference genome. By default the application will seek reads that can be aligned to an up-to-date collection of HPV reference genomes assembled by PaVE^5, 6^ in FASTA format (*2*23 reference HPV genomes and 222 non-reference HPV genomes as of 01/13/2020), but can employ a user-generated set of viral genomes if so desired. Details of downloading and generating these reference genomes can be found at the website of the HPV-EM tool. As shown in Fig. 1A, the HPV-EM tool is comprised of the following steps: (I) Align all RNA-seq reads to the human reference genome using STAR2^13^, retaining the non-aligned (non-human) reads; (II) Filter low-complexity reads using the DUST algorithm^14^, retaining all other reads; (III) Align the retained reads from the previous steps to the set of HPV reference genomes using STAR2^13^; (IV) Collect features from the aligned HPV reads, including whether a read is uniquely or ambiguously aligned, the HPV genotypes a read is aligned to, and the number of mismatches and read length; (V) Use the EM algorithm we designed (See the “Design of the EM algorithm” section below) to calculate the probability a read arose from each HPV genotype and the makeup of HPV genotypes; (VI) Assign HPV reads to the proper HPV genotypes and visualize gene expression for all the HPV genotypes estimated to be present in the sample (default threshold: 1.5 HPV transcripts per million reads). The output of the HPV-EM tool contains: a pie chart that shows the maximum likelihood estimate of the makeup of HPV genotypes, coverage maps of the HPV genotypes present in the sample, a table in TSV format that summarizes the percentages of the HPV genotypes present in the sample (including number of HPV reads for each HPV genotype, estimated sequencing error rate, total number of reads sequenced from the sample), and a table in TSV format that summarizes the HPV gene expression in each HPV genotype present in the sample.

### Design of the EM algorithm

As discussed above, the set of RNA-seq reads aligned to one or more HPV genomes is passed to an expectation–maximization (EM) algorithm in order to produce a maximum likelihood estimate (MLE) of HPV genotypes in the sample.

The EM algorithm takes an iterative approach to the problem of assigning *m* reads, denoted as ${\text{X}} = \left( {{\text{x}}^{1} , \ldots ,{\text{x}}^{{\text{i}}} , \ldots ,{\text{x}}^{{\text{m}}} } \right)$, among *n* HPV genotypes (${\text{HPV}}_{{\text{k}}}$). Define latent variables ${\text{y}}_{{\text{k}}}^{{\text{i}}}$ to have value 1 in the random event that read ${\text{x}}_{{\text{i}}}$ originates from ${\text{HPV}}_{{\text{k}}}$ and 0 if not; $Y$ is the set of all ${\text{y}}_{{\text{k}}}^{{\text{i}}}$. Further define a set of parameters $\uptheta = \left\{ {\phi_{{\text{k}}} ,\epsilon} \right\}$, where $\phi_{{\text{k}}}$ is the probability a read comes from HPV genotype *k* and $\epsilon$ is the per-base error rate of mapped HPV reads (Figure S1). The EM algorithm produces an MLE for parameters ${\uptheta }$ by first calculating the expected value of $Y$ based on the current parameter values in an E-step, then solving for ${\uptheta }$ that maximizes the value of a likelihood function in an M-step.

The log-likelihood of observing a set of reads is

$${\text{l}}\left( {\uptheta } \right) = \log {\text{p}}\left( {{\text{X}};{\uptheta }} \right) = \mathop \sum \limits_{{{\text{i}} = 1}}^{{\text{m}}} \log {\text{p}}\left( {{\text{x}}^{{\text{i}}} ;{\uptheta }} \right) = \mathop \sum \limits_{{{\text{i}} = 1}}^{{\text{m}}} \log \mathop \sum \limits_{{{\text{k}} = 1}}^{{\text{n}}} {\text{p}}\left( {{\text{x}}^{{\text{i}}} ,{\text{y}}_{{\text{k}}}^{{\text{i}}} ;{\uptheta }} \right).$$

Note that

1 $${\text{p}}\left( {{\text{x}}^{{\text{i}}} ,{\text{y}}_{{\text{k}}}^{{\text{i}}} ;{\uptheta }} \right) = {\text{p}}\left( {{\text{x}}^{{\text{i}}} \,{|}\,{\text{y}}_{{\text{k}}}^{{\text{i}}} ;\epsilon} \right){\text{p}}\left( {{\text{y}}_{{\text{k}}}^{{\text{i}}} ;\phi } \right) = {\text{c}}_{{\text{k}}}^{{\text{i}}} \phi_{{\text{k}}}$$

where

$${\text{c}}_{{\text{k}}}^{{\text{i}}} = {\text{p}}\left( {{\text{x}}^{{\text{i}}} \,{|}\,{\text{y}}_{{\text{k}}}^{{\text{i}}} ;\epsilon} \right) = \left\{ {\begin{array}{*{20}l} {\left( {1 - \epsilon} \right)^{{{\text{Lm}}_{{\text{k}}}^{{\text{i}}} }} \epsilon^{{{\text{Le}}_{{\text{k}}}^{{\text{i}}} }} } \hfill & {if\, read\, x_{{\text{i}}} \, is\,mapped\,to\,HPV_{k} } \hfill \\ {{\text{p}}_{{\text{s}}} } \hfill & {if\, read\, x_{{\text{i}}} \, is\,not\,mapped\,to\,HPV_{k} } \hfill \\ \end{array} } \right. .$$

Here, $Lm_{k}^{i}$ is the number of bases from read $i$ that match the sequence of the $HPV_{k}$ genome to which it was aligned and $Le_{k}^{i}$ is the number of mismatches. $p_{{\text{s}}}$ is a very small probability that a transcript arose from an HPV genotype to which it is not mapped.

#### E-step

In the expectation step, the algorithm defines the expected log-likelihood function $Q\left( {{\uptheta };{\uptheta }^{\left( t \right)} } \right)$ with conditional distribution over the latent variables $Y$, given reads $X$ and parameters ${\uptheta }^{\left( t \right)} = \left\{ {\phi_{k}^{\left( t \right)} ,\epsilon^{\left( t \right)} } \right\}$:

$${\text{Q}}\left( {{\uptheta };{\uptheta }^{{\left( {\text{t}} \right)}} } \right) = {\text{E}}_{{{\text{Y}}|{\text{X}};{\uptheta }^{{\left( {\text{t}} \right)}} }} \left[ {{\text{l}}\left( {\uptheta } \right)} \right] = \mathop \sum \limits_{{{\text{i}} = 1}}^{{\text{m}}} \mathop \sum \limits_{{{\text{k}} = 1}}^{{\text{n}}} {\text{p}}\left( {{\text{y}}_{{\text{k}}}^{{\text{i}}} \,{|}\,{\text{x}}_{{\text{i}}} ;{\uptheta }^{{\left( {\text{t}} \right)}} } \right)\log {\text{p}}\left( {{\text{x}}^{{\text{i}}} ,{\text{y}}_{{\text{k}}}^{{\text{i}}} ;{\uptheta }} \right)$$

Recalling (1), this can be written as

2 $${\text{Q}}\left( {{\uptheta };{\uptheta }^{{\left( {\text{t}} \right)}} } \right) = \mathop \sum \limits_{{{\text{i}} = 1}}^{{\text{m}}} \mathop \sum \limits_{{{\text{k}} = 1}}^{{\text{n}}} {\text{p}}\left( {{\text{y}}_{{\text{k}}}^{{\text{i}}} \,{|}\,{\text{x}}^{{\text{i}}} ;{\uptheta }^{{\left( {\text{t}} \right)}} } \right)\log \left( {{\text{c}}_{{\text{k}}}^{{\text{i}}} \phi_{{\text{k}}} } \right) = \mathop \sum \limits_{{{\text{i}} = 1}}^{{\text{m}}} \mathop \sum \limits_{{{\text{k}} = 1}}^{{\text{n}}} \left( {{\text{p}}\left( {{\text{y}}_{{\text{k}}}^{{\text{i}}} \,{|}\,{\text{x}}^{{\text{i}}} ;{\uptheta }^{{\left( {\text{t}} \right)}} } \right)\log {\text{c}}_{{\text{k}}}^{{\text{i}}} + {\text{p}}\left( {{\text{y}}_{{\text{k}}}^{{\text{i}}} \,{|}\,{\text{x}}^{{\text{i}}} ;{\uptheta }^{{\left( {\text{t}} \right)}} } \right)\log \phi_{{\text{k}}} } \right) .$$

#### M-step

In the maximization step, the estimates for the model parameters are updated to the next iteration ${\uptheta }^{{\left( {t + 1} \right)}}$ by maximizing $Q\left( {{\uptheta };{\uptheta }^{\left( t \right)} } \right)$ in (2). Define $A_{k}$ as the expected number of reads that originate from $HPV_{k}$ under ${\uptheta }^{\left( t \right)}$. Then,

$${\text{A}}_{{\text{k}}} = \mathop \sum \limits_{{{\text{i}} = 1}}^{{\text{m}}} {\text{p}}\left( {{\text{y}}_{{\text{k}}}^{{\text{i}}} \,{|}\,{\text{x}}^{{\text{i}}} ;{\uptheta }^{{\left( {\text{t}} \right)}} } \right) = \mathop \sum \limits_{{{\text{i}} = 1}}^{{\text{m}}} \frac{{{\text{p}}\left( {{\text{x}}^{{\text{i}}} ,{\text{y}}_{{\text{k}}}^{{\text{i}}} ;{\uptheta }^{{\left( {\text{t}} \right)}} } \right)}}{{{\text{p}}\left( {{\text{x}}^{{\text{i}}} ;{\uptheta }^{{\left( {\text{t}} \right)}} } \right)}} = \mathop \sum \limits_{{{\text{i}} = 1}}^{{\text{m}}} \frac{{{\text{c}}_{{\text{k}}}^{{\text{i}}} \phi_{{\text{k}}}^{{\left( {\text{t}} \right)}} }}{{\mathop \sum \nolimits_{{{\text{j}} = 1}}^{{\text{n}}} {\text{c}}_{{\text{j}}}^{{\text{i}}} \phi_{{\text{j}}}^{{\left( {\text{t}} \right)}} }} .$$

Update rules for ${\uptheta }$ to maximize $Q\left( {{\uptheta };{\uptheta }^{\left( t \right)} } \right)$ are defined by taking derivatives with respect to each parameter and setting them equal to zero. Defining $w_{k}^{i} = p\left( {y_{k}^{i} \,{|}\,x^{i} ;{\uptheta }^{\left( t \right)} } \right)$, we then have

$$\begin{aligned} & \nabla_{{\phi_{{\text{k}}} }} {\text{Q}}\left( {\phi_{{\text{k}}} ,\epsilon;{\uptheta }^{{\left( {\text{t}} \right)}} } \right) = 0 \to \phi_{{\text{k}}}^{{\left( {{\text{t}} + 1} \right)}} = \frac{{{\text{A}}_{{\text{k}}} }}{{\text{m}}} \\ & \nabla_{\epsilon} {\text{Q}}\left( {\phi_{{\text{k}}} ,\epsilon;{\uptheta }^{{\left( {\text{t}} \right)}} } \right) = 0 \to \epsilon^{{\left( {{\text{t}} + 1} \right)}} = \left( {\frac{{\mathop \sum \nolimits_{{{\text{i}} = 1}}^{{\text{m}}} \mathop \sum \nolimits_{{{\text{k}} = 1}}^{{\text{n}}} {\text{w}}_{{\text{k}}}^{{\text{i}}} {\text{Lm}}_{{\text{k}}}^{{\text{i}}} }}{{\mathop \sum \nolimits_{{{\text{i}} = 1}}^{{\text{m}}} \mathop \sum \nolimits_{{{\text{k}} = 1}}^{{\text{n}}} {\text{w}}_{{\text{k}}}^{{\text{i}}} {\text{Le}}_{{\text{k}}}^{{\text{i}}} }} + 1} \right)^{ - 1} .\\ \end{aligned}$$

The E- and M-steps are then iterated until the value of $Q\left( {{\uptheta };{\uptheta }^{\left( t \right)} } \right)$ converges.

HPV-EM runs the EM algorithm multiple times (default: 5) from randomly generated initial values in order to maximize the chance of convergence to the global likelihood maximum, selecting the result with the highest likelihood value. HPV-EM then reports the MLE read counts and probabilities for the HPV genotypes detected within a sample, if any.

### Use cases of the EM algorithm

Due to repetitive and homologous sequences, RNA-seq reads from one sample can often be aligned to multiple HPV genotypes at the same time. The HPV-EM algorithm calculates the maximum likelihood estimate for the proportion of each HPV genotype in a sample. If the sample only contains one HPV genotype, then the algorithm will predict the sample to be 100% of this HPV genotype. If the sample is a mixture of different HPV genotypes, the algorithm will predict the probablity of all the HPV genotypes presented. For example, Figs. 1B–G show RNA-seq reads attributed to various genotypes in two cervical cancer samples from TCGA-CESC cohort. Sample 1 (TCGA-4J-AA1J) has RNA-seq reads aligned to 3 different HPV genotypes, namely HPV18, HPV45, and HPV97 (Fig. 1B). Sample 2 (TCGA-C5-A3HF) has RNA-seq reads aligned to 7 different HPV genotypes, namely HPV16, HPV18, HPV33, HPV45, HPV58, HPV77, and HPV195 (Fig. 1E). The application of HPV-EM increases the likelihood of correctly estimating whether samples contain a single HPV genotype or multiple HPV genotypes. After running the HPV-EM tool, Sample 1 is estimated by the EM algorithm to contain 100% HPV18 (Fig. 1C), while Sample 2 is estimated to contain both HPV18 and HPV58 (Fig. 1F). The HPV coverage map of Sample 1 is shown in Fig. 1D, which only includes HPV18, and it shows a clear HPV integration pattern at the HPV18 E2 gene. The HPV coverage maps of Sample 2 are shown in Fig. 1G, which include both HPV18 and HPV58 with different levels of HPV read coverage.

### Evaluation using cell line data

We tested the ability of HPV-EM to accurately detect various levels of HPV gene expression and genotypic makeups for samples with single and mixed HPV genotypes by creating a series of simulated data sets using real cervical cancer cell line data. We first downloaded paired-end RNA-seq data for SiHa (HPV16; SRR6031946) and HeLa (HPV18; SRR7276617) cells from the NCBI SRA database and validated their HPV genotypes using HPV-EM. We then retained the HPV reads from the two cervical cancer cell line data sets by running Steps (I)–(III) of the HPV-EM tool (Refer to the section of “Overview of the HPV-EM tool” section above), substituting BWA^15^ for STAR2 in Step III in order to obtain the set of HPV-aligned reads from an independent source. Note that these retained HPV reads still contain repeats that can be aligned to multiple HPV genotypes. We randomly selected a TCGA-CESC sample (TCGA-HM-A6W2) designated by TCGA consortium study^16^ as HPV-negative, which contains not only human RNA-seq reads but also all other types of reads generated by the current sequencing technology. To accurately spike-in HPV-reads to the intended HPV expression levels, RNA-seq reads in the above three data sets were trimmed uniformly to 48 nt to match the read length of TCGA-CESC samples. We spiked-in SiHa (i.e., HPV16) and HeLa (i.e., HPV18) reads to the HPV-negative sample, at the overall HPV Transcripts Per Million (TPM) levels 0.2, 0.5, 1, 2, 5, 10, 100, and 1,000. For each TPM level, HPV16 and HPV18 reads were randomly selected with the ratios of 0:100, 1:99, 10:90, 25:75, 50:50, 75:25, 90:10, 99:1, and 100:0. Note that some levels (e.g., TPM = 0.2 and HPV16:HPV18 = 1:99) represent less than 1 HPV read and therefore were omitted from the simulation. These simulated samples were processed by HPV-EM using standard settings, except that the TPM threshold was set to 0 to report the presence of HPV reads in all the above abundances (e.g. TPM values 0.2 and 0.5). The HPV-EM estimated TPM values and HPV16:HPV18 ratios for each sample were then compared with the known true values and ratios for each simulated sample.

### Benchmarking using TCGA-CESC data

In order to assess the performance of our HPV-EM tool on real data sets, we performed HPV genotyping for the full provisional TCGA cervical cancer cohort of 304 primary tumour samples with paired-end whole transcriptome sequencing data. A subset of 178 of these patients were previously included in the TCGA-CESC consortium study, and the HPV genotypes of these patients were detected using both MassArray and RNA-seq data, with a subset also having whole genome sequencing data^16^. Using the HPV genotyping result from TCGA consortium study as a gold standard, we evaluated the accuracy of the HPV-EM tool in HPV genotyping using only RNA-seq data. To evaluate the performance of HPV-EM compared with existing tools, we searched and found several HPV or virus genotyping tools that take RNA-seq data as input. Among these tools, HPVDetector^9^ is the most contemporary and dedicated HPV genotyping tool. We also intended to run other virus genotyping tools using TCGA-CESC data, including VirusTAP^17^ and Vipie^18^. However, neither of these tools was suitable for this task. VirusTAP, which runs via a web user interface, imposes a maximum file size of 10 GB for uploaded gzipped FASTQ files, making it unable to handle 24% of TCGA-CESC RNA-seq files. Vipie was also intended to be run via a web user interface, however at the time of testing, its website was offline. Therefore, we compared HPV-EM and HPVDetector on the 178 TCGA-CESC samples with the HPV genotyping result from TCGA consortium study. For this experiment, TCGA-CESC RNA-seq data were downloaded from the GDC file repository^19^ in BAM format. Raw reads were extracted from the BAMs in FASTQ format and used as input for executions of HPV-EM and HPVDetector, each with default parameters. The human reference genome used with these tools was GRCh38 (r90).

### Validation using DNA tiling array data

As an additional validation, we executed HPV-EM on RNA-seq data from a cohort of cervical cancer patients treated at Washington University School of Medicine. Cervical tumour samples were collected as part of a prospective tumour banking study (201105374), and data analyses were performed as part of a retrospective analysis study with waiver of consent (202001180). Approval of both studies of Washington University Institutional Review Board (IRB) and waiver of consent of 202001180 by Protocol Review and Monitoring Committee (PRMC) of Washington University was obtained. All methods were carried out in accordance with all relevant guidelines and regulations. These patients were uniformly treated with curative-intent chemoradiation therapy and clinical outcome data were prospectively collected. RNA-seq data (*N* = 67) were generated in the Genome Technology Access Center (GTAC) at Washington University (Illumina HiSeq 2500, 1 × 50 nt, ~ 40 million reads per sample). HPV genotyping by DNA tiling array was also performed for an expanded cervical cancer cohort of 90 patients (including the 67 with RNA-seq data) and the relationship between HPV genotypes and patient prognosis was examined using prospectively gathered patient clinical information. The DNA tiling array experiment probes were designed for 33 HPV genotypes, covering the whole genomes of HPV16 and HPV18 and the E6 and E7 regions of the other 31 HPV genotypes, as described previously^20, 21^. On average, 156,114 reads were acquired by the probes for each sample and sequenced using the Illumina HiSeq platform with paired 2 × 100 bp reads, and a threshold of 100 reads was used as the cut off to accurately call an HPV genotype. The HPV genotype results from the tiling array were compared to those generated by HPV-EM among the 67 patients with RNA-seq data. Since HPVDetector is not capable of analysing single-end sequencing data, only HPV-EM was executed and compared with DNA array data.

### Application to TCGA-HNSC data

To demonstrate the broader applicability of our HPV-EM tool, we ran HPV-EM on the full TCGA-HNSC provisional cohort of 499 head and neck primary tumour samples with paired-end RNA-seq data. We benchmarked our analysis based on the TCGA-HNSCC consortium study^22^, which included a subset of 279 samples and identified 36 HPV-positive HNSCC cases. Using the full provisional cohort, we demonstrated the HPV genotypic differences in patient prognosis in head and neck squamous cell cancers. For these experiments, raw sequencing data were downloaded from GDC and patient clinical data were obtained from TCGAbiolinks^23^.

## Results

### Evaluation using cell line data

**Figure 2:**
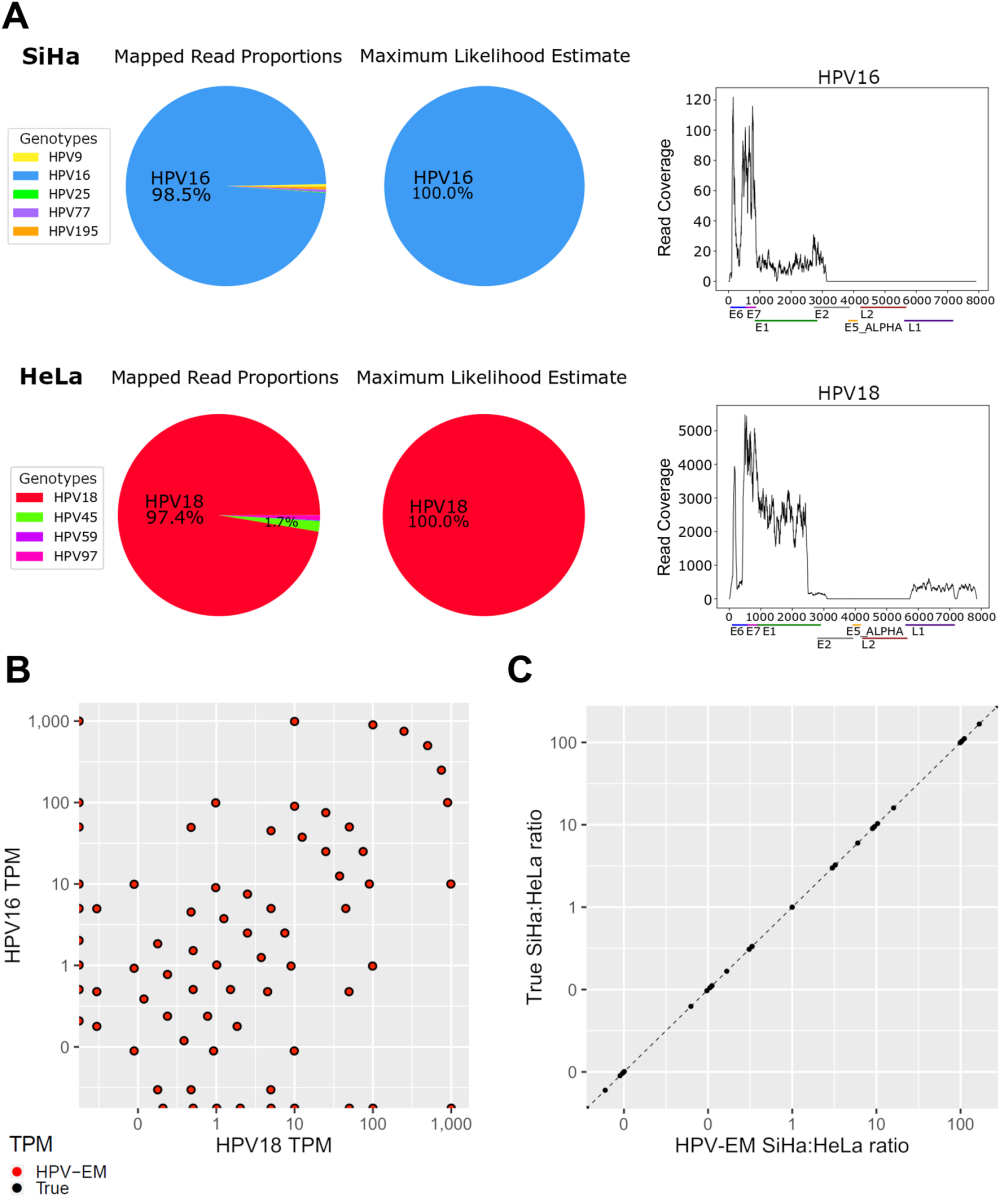
Evaluation of HPV-EM using cell line and simulated data. (**A**) HPV-EM validated SiHa cells are HPV16-positive and HeLa cells are HPV18-positive. Due to homology between genotypes, a small proportion of reads from each cell line sample aligned to alternative HPV genotypes; however, after expectation maximization, HPV-EM correctly identifies both cell line samples as containing only a single HPV genotype. (**B**) Comparison of HPV-EM’s predicted levels of HPV16 and HPV18 expression to the true levels in the set of simulated samples. HPV18 expression in TPM is denoted on the x-axis, HPV16 expression in TPM on the y-axis, the per-sample expression reported by HPV-EM is marked in red, and the true levels of expression are marked in black. (**C**) Comparison of HPV-EM results to true HPV16 and HPV18 ratios in the set of simulated samples. The x-axis denotes the ratio of SiHa to HeLa HPV reads (HPV16:HPV18) reported in each sample by HPV-EM and the y-axis denotes the true ratio. The fitted curve has an ${\varvec{R}}^{2}$ value of 1, representing the near-perfect performance of HPV-EM in detecting HPV reads in these samples.

To assess the accuracy of HPV-EM in detecting HPV transcription and estimating HPV genotypic makeups at various levels of viral gene expression, we ran HPV-EM on a set of simulated samples derived from spike-ins of SiHa-derived HPV16 reads and HeLa-derived HPV18 reads into a known HPV-negative sample (see the section on “Evaluation using cell line data” in “Materials and methods” section). First, we intended to use HPV-EM to validate the HPV genotypes for the RNA-seq data from SiHa and Hela cells. While both samples include a small amount (< 5%) of repetitive and homologous reads that could be aligned to multiple HPV genotypes, our HPV-EM tool is able to accurately predict the HPV genotypic makeups of the samples (Fig. 2A). Consistent with the known HPV genotypes of these samples, HPV-EM estimated reads from SiHa cells as 100% HPV16 and reads from HeLa cells as 100% HPV18 (Fig. 2A). Next, we intended to use the simulated spike-in data sets to examine whether HPV-EM is able to accurately detect the presence of HPV at various gene expression levels and HPV genotypic ratios. Figure 2B shows the spiked-in TPM values of HPV16 and HPV18 gene expression (black dots), together with the HPV-EM estimated TPM values of the two HPV genotypes (red dots). We can see that the HPV-EM tool demonstrated excellent specificity across 5 orders of magnitude of HPV expression (TPM from 0.01 to 1,000), returning no false positives in any test case (Fig. 2B). On average, the HPV-EM tool detected 100% of the total spiked-in HPV16 reads and 99.9% of the total spiked-in HPV18 reads in these samples (Table S1). Figure 2C shows the spiked-in HPV16:HPV18 ratios (x-axis) and the HPV-EM estimated HPV16:HPV18 ratios (y-axis) for the simulated data sets. We can see that the HPV-EM tool demonstrated excellent specificity in estimating HPV genotypic makeups at all the levels of HPV gene expression tested (Pearson correlation, R^2^ = 1; Fig. 2C).

### Benchmarking using TCGA-CESC data

#### Optimized assignment of HPV reads

**Figure 3:**
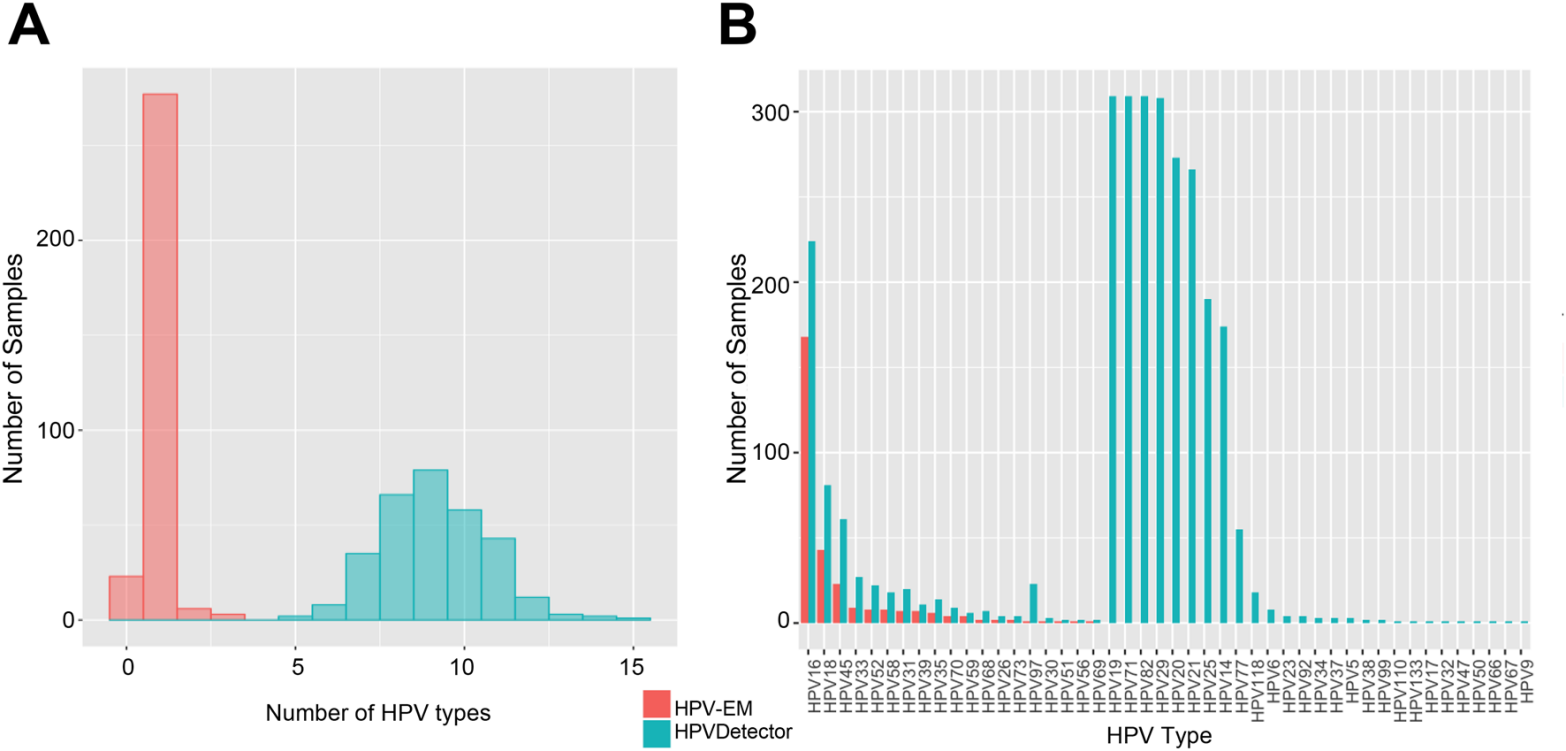
Optimization of HPV reads assignment by the EM algorithm. (**A**) Histogram of the number of HPV genotypes reported per sample by HPV-EM and HPVDetector when analysing RNA-seq data from 304 samples of TCGA-CESC cohort. The number of samples reported as containing each number of HPV genotypes by HPV-EM is plotted in red, the number reported by HPVDetector is plotted in teal. (**B**) Histogram of the number of TCGA-CESC samples reported as containing each HPV genotype by HPV-EM and HPVDetector. The number of samples reported as for each by HPV-EM is plotted in red and the number by HPVDetector in teal. HPV-EM’s predictions for the TCGA-CESC cohort are 97% consistent with those of TCGA-CESC consortium study (2017) among the 178 samples for which they performed HPV genotyping. This demonstrates HPV-EM’s advantage in effectively assigning the repetitive reads to the real high-risk HPV genotypes present in the samples.

To test whether HPV-EM can effectively remove false HPV genotyping calls caused by repetitive sequencing reads and homologies, we applied our tool to 304 primary cervical cancer samples from TCGA-CESC cohort that have full transcriptome sequencing data available (Table S2). As a comparison, we also analysed the same data set using a contemporary HPV genotyping tool, HPVDetector^9^, which supports whole transcriptome sequencing data. HPV-EM identified 285 (94%) of the 304 primary cervical cancer samples as HPV-positive. Within the 285 HPV-positive samples, 275 (96.5%) have 1 HPV genotype, 8 (2.8%) have 2 HPV genotypes, and only 2 (0.7%) have 3 HPV genotypes. HPVDetector identified 304 (100%) of the primary cervical cancer samples as HPV-positive. Moreover, the HPVDetector identified all 304 samples as having at least low levels of 3 or more (up to 15) HPV genotypes. In Fig. 3A, we present histograms of the number of HPV genotypes per sample estimated by HPV-EM and HPVDetector. For the HPV-positive cases, HPV-EM estimated on average 1.04 ± 0.23 HPV genotypes are present in a sample, which is significantly below the estimate of 9.14 ± 1.59 produced by HPVDetector (Fig. 3A). Since previous studies using array methods^8, 16^ showed that the majority of HPV infections are from a single HPV genotype, this result demonstrates that our EM algorithm effectively removed false HPV genotyping calls caused by repetitive sequencing reads and homologies. In Fig. 3B, we show the number of samples predicted to contain each HPV genotype by HPV-EM and HPVDetector among the 304 TCGA-CESC samples. HPV-EM identified 19 HPV genotypes in the TCGA-CESC cohort (*N* = 304), encompassing the previously identified high-risk HPV genotypes. Among these, HPV16 is the most frequent HPV genotype (167 out of 304, or 55%), and HPV18 is the second frequent HPV genotype (43 out of 304, or 14%). The remaining samples consist of HPV genotypes including HPV45, HPV33, HPV52, HPV58, HPV31, etc. (Fig. 3B). On the other hand, HPVDetector reported HPV19, HPV71, and HPV82 as the most frequent HPV genotypes in all the 304 samples, with 23 additional HPV genotypes that have not been previously reported as causing cervical cancer (Fig. 3B). This analysis demonstrates HPV-EM’s advantage in effectively assigning the repetitive reads aligned to multiple HPV genotypes to the high-risk HPV genotypes present in the samples.

#### Comparison with TCGA consortium study

**Figure 4:**
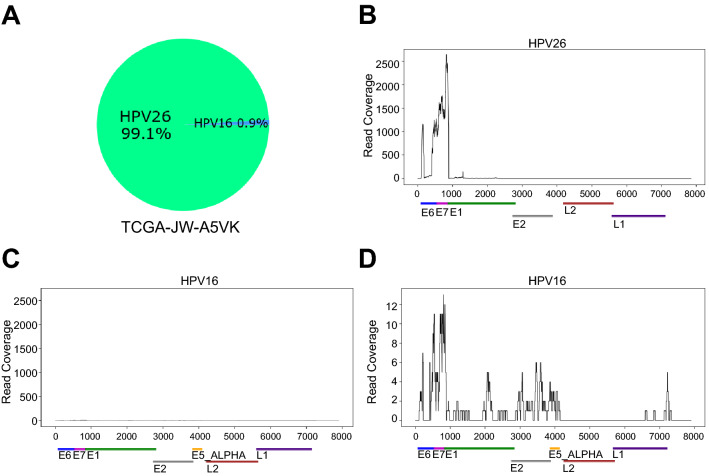
Maximum likelihood estimates of HPV genotypes and read coverage for TCGA-JW-A5VK. (**A**) Maximum likelihood estimate of the proportions of HPV genotypes that are present in TCGA-JW-A5VK using HPV-EM. (**B**) Read coverage of HPV26 in TCGA-JW-A5VK. (**C**) Read coverage of HPV16 in TCGA-JW-A5VK using the same scale as in panel B to compare with HPV26. (**D**) A close view of HPV16 read coverage in TCGA-JW-A5VK. Our analysis agrees with the TCGA consortium study on cervical cancer (2017) in that a mixture of HPV16 and HPV26 reads are present in the sample; however, whereas TCGA assign this case a final type of HPV16 based on hierarchical attribution, HPV-EM reveals that HPV26 is by far the dominant HPV genotype in this sample, with a < 1% chance of reads arising from HPV16. Decreased read coverage after E7 of HPV26 represents HPV26 integration to the human genome, whereas HPV16 has low read coverage throughout the HPV16 viral genome.

To further estimate the accuracy of HPV-EM, we identified a subset (*N* = 178) of the primary cervical cancer samples that were included in TCGA consortium study^16^. For these 178 samples, both RNA-seq and MassArray data were corroborated and compared using TCGA’s in-house strategies to decide the final HPV genotypes. The HPV-EM tool only used RNA-seq data to detect and estimate the HPV genotypes in the same set of samples. In the TCGA consortium study, 169 out of the 178 cervical cancer samples were estimated to be HPV-positive (166 with 1 HPV genotypes as the final genotype, and 3 with a second genotype). While HPV-EM identified the same 169 samples as HPV-positive, we compared the HPV genotyping calls against TCGA results. HPV-EM’s prediction is 97% consistent (i.e. 164 out of 169) with the result of TCGA consortium study as a gold standard. The 5 cases of disagreement between HPV-EM and TCGA genotyping calls are presented in Table S2, with the level of RNA-seq read support following each HPV-EM call. In each case TCGA predicted only a single HPV genotype and HPV-EM predicted multiple types, with the HPV genotype with the greatest gene expression matching that detected by TCGA; however, the secondary HPV genotypes predicted by HPV-EM, while present at a lower level, still have significant read support (from 116 to 1,248 pairs of reads; Table S2). In one additional case (TCGA-JW-A5VK), TCGA consortium study called this sample as having a joint HPV16 and HPV26 infection, with HPV16 as the primary and HPV26 as a secondary infection based on a hierarchical attribution method used in the study. However, HPV-EM revealed that there is significant HPV26 gene expression (99%; 10,552 pairs of reads) in the sample compared to HPV16 gene expression (1%; 97 pairs of reads) (Fig. 4). This implies a potential designation of HPV26 as a “high-risk” HPV genotype, suggesting that HPV26 in the A5 group may be driving this cervical cancer case similarly to HPV16 in the A9 and HPV18 in the A7 group.

### Validation using DNA array

**Figure 5:**
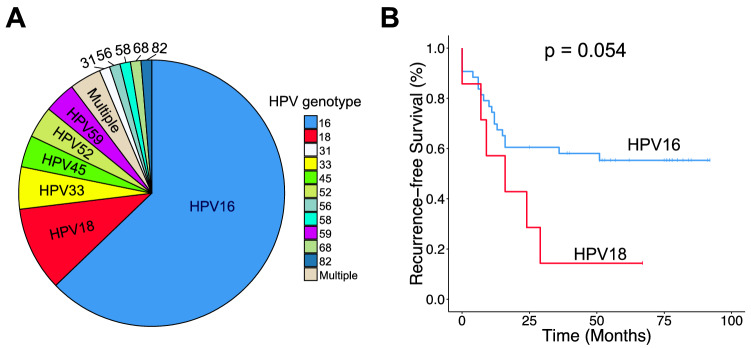
HPV genotype distribution and association with CRT outcome. (**A**) HPV genotype distribution with the expanded Washington University institutional cervical cancer cohort: 49 cases of HPV16, 8 cases of HPV18, and 33 cases of another or multiple genotypes. (**B**) Kaplan–Meier analysis shows that patient outcome is associated with HPV genotype. The samples consist of an institutional cervical cancer cohort (*N* = 90) of patients uniformly treated with chemoradiation therapy. All samples were genotyped using DNA tiling array data, including 67 with RNA-seq data. The survival outcomes of 50 patients with either HPV16 or HPV18 are plotted; HPV18-positive patients demonstrate significantly worse recurrence-free survival outcomes than those with HPV16.

To evaluate whether using RNA-seq data alone is sufficient for accurate HPV genotyping of tumour samples, we next applied HPV-EM to a cohort of 67 institutional primary cervical cancer samples sequenced with RNA-seq data and validated HPV-EM’s predictions using DNA tiling array. HPV-EM detected HPV gene expression in 57 (85%) of the 67 samples using RNA-seq data (Table S3). Within the 57 HPV-positive samples, 52 (91%) have 1 HPV genotype and 5 (8.8%) have 2 or more HPV genotypes (Table S3). Comparing HPV-EM’s predictions with those of the DNA array data, 56 out of 57 cases with HPV gene expression detected by HPV-EM were confirmed by the DNA array data to be HPV-positive, 9 out of 10 HPV-negative cases were also confirmed by the DNA array data. Therefore, HPV-EM achieved an 96.5% of accuracy in detecting HPV gene expression using DNA array as a gold standard. Within the 57 HPV-positive cases, 52 (91%) were predicted to be completely consistent with DNA array data and 1 (1.8%) was predicted by the tiling array to be HPV-negative (Table S3). Of the remaining 4 cases, in 3 the primary HPV genotype predicted by HPV-EM matches that detected by the DNA array. The last case was identified by the DNA array data as HPV16 and HPV66, but identified by HPV-EM to have HPV44 (8,377 RNA-seq reads) and HPV16 (3,007 RNA-seq reads). This is because the HPV44 genotype was not included in the probe design of the DNA array. At this point, we have corroborated HPV genotyping using RNA-seq and DNA array data, and we intended to explore whether HPV genotypes are associated to cervical cancer patient outcomes after chemoradiation therapy (CRT). Since HPV16 and 18 are the most frequent HPV genotypes, we used the Kaplan–Meier estimator to compare recurrence-free survival (RFS) after CRT of cervical cancer patients with the two HPV genotypes. Patients with HPV18 show unfavourable RFS outcomes compared with those with HPV16 (Logrank test, *p* = 0.054) (Fig. 5).

### Application to in TCGA-HNSC data

**Figure 6:**
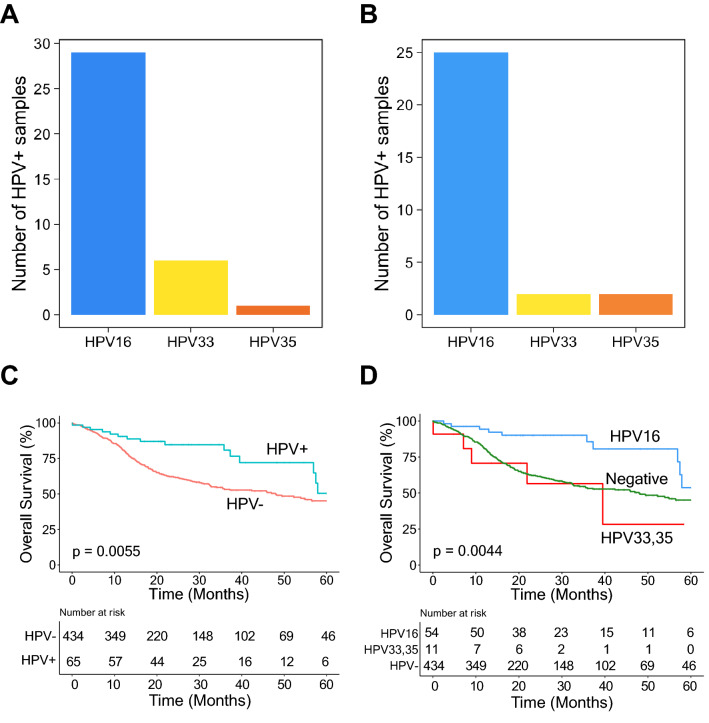
HPV genotypes and their genotypic association to patient outcomes in TCGA-HNSC data. (**A**) Genotype distribution among HPV-positive tumours in the 279 cases analysed in TCGA-HNSC consortium study. HPV-EM was able to accurately identify 100% of the HPV-positive cases. (**B**) Genotype distribution among HPV-positive tumours in the 220 additional cases in the TCGA-HNSC provisional cohort. The distribution and percentage of HPV genotypes is the same as the TCGA-HNSC consortium study. (**C**) Kaplan–Meier analysis suggests that HPV-positive head-and-neck patients (when combined as a group) have better outcomes than HPV-negative head-and-neck patients. (**D**) Separating HPV16-positive patients from HPV33- and HPV35-positive patients shows that HPV16-positive patients have better survival outcomes, while HPV33- and HPV35-positive patients have inferior survival outcomes compared to HPV16 and HPV-negative patients.

To demonstrate its broader applicability, we applied HPV-EM to TCGA head and neck cohort (TCGA-HNSC). In the TCGA-HNSC consortium study, 279 of these head and neck cancer samples were included, and 36 among these samples were identifed in the study as HPV-positive. HPV-EM’s HPV genotyping predictions (Table S4) were 100% consistent with the TCGA study in these 36 (13.9%) HPV-positive cases (Fig. 6A). The 36 samples include 29 HPV16 cases, 6 HPV33 cases, and 1 HPV35 case. We further applied the HPV-EM tool to the addtional 220 TCGA-HNSC samples in the current provisional data set available from GDC, and HPV-EM discovered an additional 29 (13.2%) HPV-positive samples (Table S4). These 29 samples include 25 HPV16 cases, 2 HPV33 cases, and 2 HPV35 cases (Fig. 6B). As expected, the distribution of the HPV genotypes from the additional data set is not statistically different from the data set used in the previous TCGA study, indicating that HPV-EM is consistently accurate using different data sets. Since HPV status (i.e. HPV-positive and HPV-negative) has previously been associated to head and neck cancer patient outcomes, we first compared all the above 65 HPV-positive samples (i.e. 36 + 29) with all other 434 HPV-negative samples (Fig. 6C). It seemed that the combined HPV16, HPV33, and HPV35 patient cohort has favorable outcomes compared to the patients with HPV-negative cancers (*p* = 0.0055). However, when breaking down HPV-positive patients by genotypic group, we can see that the combined HPV33 and HPV35 group has unfavorable outcomes compared to both the HPV16 group and HPV-negative patients (*p* = 0.0044) (Fig. 6D).

## Discussion

Overall, we designed and implemented a novel tool, designated HPV-EM, for highly sensitive and accurate HPV detection and genotyping using whole transcriptome sequencing data. To address the ambiguities caused by repetitiveness and homology in sequencing data, we designed and implemented a novel expectation–maximization (EM) algorithm in our tool to calculate an optimized mapping of sequencing reads to HPV reference genomes based on a probability model. We validated the sensitivity and accuracy of our tool using simulated data, gold standards in real public data, and DNA tiling array data. In addition to HPV detection and genotyping, the HPV-EM tool also provides functions to estimate and visualize HPV gene expression, which will aid researchers with their functional studies related to HPV biology. An HPV genotyping tool without limitations in HPV detection and genotyping is crucial in facilitating epidemiology studies and vaccine trials, as well as basic research in HPV-related diseases.

The EM algorithm we designed in the HPV-EM tool is essential to accurately identify whether a sample contains a single or multiple HPV genotypes and to identify the proportions of each genotype. Because of repeats, homology and sequencing errors, HPV reads from a sample can be aligned to multiple HPV reference genomes, as represented in the samples shown in Fig. 1. The EM algorithm calculates the likelihood of the identified HPV genotypes based on a probability model and eventually converges to a highly accurate estimate of the HPV genotypes and their makeup present in the sample. When used to analyze both the institutional and publicly available data sets, the HPV-EM tool accurately identified the real cases with a mixture of HPV genotypes from the cases with single HPV genotype that may contain reads alignable to multiple HPV genotypes due to the factors mentioned above. This can help improve the accuracy in ongoing studies comparing the biological differences in HPV related cancers. This also makes it feasible to identify and study the cases with mixtures of HPV genotypes using cutting-edge technologies such as single-cell RNA sequencing. The biological and medical consequences of secondary HPV infections also warrant futher examination in other downstream analyses or experiments in HPV-related research and clinical settings.

In this study, we demonstrated HPV genotypes play an important role in treatment resistance and patient outcomes in cervical cancer. The rationale of comparing HPV genotypes in cervical cancer patient outcomes stems from the fact that virtually all cervical cancer cases can be traced to HPV infection and progression. Recent studies using outcome data from independent Chinese and Japanese cohorts showed that cervical cancer patients with HPV16 have favorable progression-free and overall survival comparing to other non-HPV16 patients^24–26^. Using HPV-EM, we accurately discovered the HPV genotypes in our institutional cervical cancer cohort treated uniformly with curative-intent chemoradiation therapy (CRT), and further demonstrated cervical cancer patients with HPV18 (as the second most prevalent HPV genotypes in cervical cancer) have worse recurrence-free survival compared to HPV16 (Fig. 5).

While our study has focused on cervical cancer, we also applied the HPV-EM algorithm to TCGA-HNSC data to benchmark our tool with the consortium study. In the head and neck, HPV status (i.e. HPV-positive and HPV-negative) is currently considered a prognostic marker, with HPV-positive head and neck cancers associated to favorable survival outcomes. Our study demonstated that while head and neck patients with HPV16 have favorable overall survival, patients with HPV33 and HPV35 have worse patient outcomes. The rich HPV genotypes shown in patients with cervical and head and neck cancers warrant further comparison between their genomic characteristics (e.g. integration, HPV gene expression, and gene regulation) to develop HPV genotype-specific treatment methods in future genomics and translational studies.

Taken together, we developed an accurate HPV detection and genotyping algorithm to identify the exact HPV genotypic makeup of HPV-positive cases and demonstrated HPV genotypes as prognostic biomarkers in both cervical and head and neck cancers. These HPV genotype-specific prognostic biomarkers could be applied to modify therapies for patients (to either escalate or de-escalate CRT treatment) according to risks of failure after CRT and improve patient outcomes. We expect HPV-EM will be applied broadly in HPV-related cancer research and clinical settings. The algorithm also has the potential to be adapted to studies in other pathogenic microbes and metagenomic studies.

## Supplementary information

Supplementary information.

## Data Availability

HPV-EM is an open source collaborative initiative available in the GitHub repository (https://github.com/jin-wash-u/HPV-EM).
