# Supplementary material for: HPV-EM: an accurate HPV detection and genotyping EM algorithm
Source: Sci Rep. 2020 Aug 31;10:14340. doi: 10.1038/s41598-020-71300-7 (PMC7459114; doi:10.1038/s41598-020-71300-7)
Supplement: Supplementary file 1 — Supplementary information. [file 41598_2020_71300_MOESM1_ESM.pdf]

Supplementary data

HPV-EM: An accurate HPV detection and genotyping EM algorithm

Matthew Inkman[1], Kay Jayachandran[1], Thomas M. Ellis[1,2], Fiona Ruiz[1], Michael D. McLellan[3], Christopher A. Miller[3], Yufeng Wu[4], Akinyemi I. Ojesina[5,6,7], Julie K. Schwarz[1,8,9] and Jin Zhang[1,8,10]*

[1] Department of Radiation Oncology, Washington University School of Medicine, St. Louis, MO, 63108, USA
[2] Department of Computer Science, Washington University in St. Louis, St. Louis, MO, 63105, USA
[3] McDonnell Genome Institute, Washington University School of Medicine, St. Louis, MO, 63108, USA
[4] Computer Science and Engineering Department, University of Connecticut, Storrs, CT, 06269, USA
[5] Department of Epidemiology, University of Alabama at Birmingham, Birmingham, AL, 35294, USA
[6] O'Neal Comprehensive Cancer Center, University of Alabama at Birmingham, Birmingham, AL, 35294, USA
[7] Hudson-Alpha Institute for Biotechnology, Huntsville, AL, 35806, USA
[8] Siteman Cancer Center, Washington University School of Medicine, St. Louis, MO, 63108, USA
[9] Department of Cell Biology and Physiology, Washington University School of Medicine, St. Louis, MO, 63108, USA
[10] Institute for Informatics, Washington University School of Medicine, St. Louis, MO, 63108, USA

* To whom correspondence should be addressed. Tel: +1 (314) 273-3278; Fax: +1 (314) 747-5498; Email: jin.zhang@wustl.edu

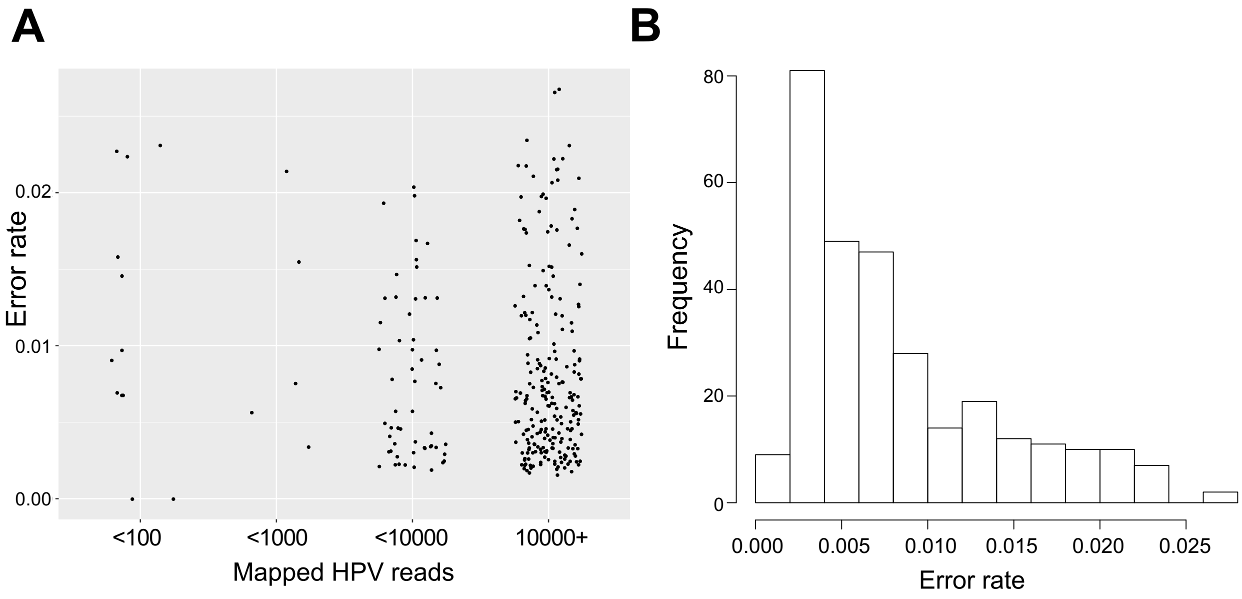

Figure S1. Maximum likelihood estimates of sequence error rate (ε) for TCGA-CESC tumor samples. **(A)** Plot of sequencing error rates by total number of HPV reads per sample. **(B)** Histogram of sequencing error rates for all HPV-positive cervical cancer samples (*N*=299). Sequencing error rates were estimated by *HPV-EM* to be in the range of 0 through ~0.025, which are in line with expected sequencing error rate from the current next-generation sequencing technologies. The variable ε could include both errors from the sequencing technologies and mutations from a specific sample. No sample was estimated with a significant high value of ε, suggesting that no highly mutated HPV samples are present in the TCGA cervical cancer cohort.

Table S1. Results of HPV-EM analysis of simulated samples. A series of simulated HPV-positive samples were prepared by spiking in HPV reads isolated from SiHa (HPV16) and HeLa (HPV18) cell line samples into an HPV-negative sample from the TCGA-CESC cohort. HPV-EM was then used to analyse these simulated samples. The results of the HPV-EM estimate of HPV expression by genotype are compared to the true values for each sample in this table.

Targeted Total HPV TPM	True HPV16 TPM	HPV-EM Reported HPV16 TPM	True HPV18 TPM	HPV-EM Reported HPV18 TPM	True HPV16 Read Count	HPV-EM Reported HPV16 Read Count	True HPV18 Read Count	HPV-EM Reported HPV18 Read Count
0.2	0	0	0.20833786	0.20833786	0	0	14	14
0.2	0.029762551	0.029762551	0.178575308	0.178575308	2	2	12	12
0.2	0.089287657	0.089287657	0.089287657	0.089287657	6	6	6	6
0.2	0.178575308	0.178575308	0.029762551	0.029762551	12	12	2	2
0.2	0.20833786	0.20833786	0	0	14	14	0	0
0.5	0	0	0.505963223	0.505963223	0	0	34	34
0.5	0.029762543	0.029762543	0.47620068	0.47620068	2	2	32	32
0.5	0.11905017	0.11905017	0.386913053	0.386913053	8	8	26	26
0.5	0.238100347	0.238100347	0.238100347	0.238100347	16	16	16	16
0.5	0.386913053	0.386913053	0.11905017	0.11905017	26	26	8	8
0.5	0.47620068	0.47620068	0.029762543	0.029762543	32	32	2	2
0.5	0.505963223	0.505963223	0	0	34	34	0	0
1	0	0	1.011925934	1.011925934	0	0	68	68
1	0.089287582	0.089287582	0.922638352	0.922638352	6	6	62	62
1	0.23810022	0.23810022	0.773825714	0.773825714	16	16	52	52
1	0.505962967	0.505962967	0.505962967	0.505962967	34	34	34	34
1	0.773825714	0.773825714	0.23810022	0.23810022	52	52	16	16
1	0.922638352	0.922638352	0.089287582	0.089287582	62	62	6	6
1	1.011925934	1.011925934	0	0	68	68	0	0
2	0	0	2.02384982	2.02384982	0	0	136	136
2	0.178574984	0.178574984	1.845274836	1.845274836	12	12	124	124
2	0.505962455	0.505962455	1.517887365	1.517887365	34	34	102	102
2	1.01192491	1.01192491	1.01192491	1.01192491	68	68	68	68
2	1.517887365	1.517887365	0.505962455	0.505962455	102	102	34	34
2	1.845274836	1.845274836	0.178574984	0.178574984	124	124	12	12
2	2.02384982	2.02384982	0	0	136	136	0	0
5	0	0	5.000084674	5.000084674	0	0	336	336
5	0.029762409	0.029762409	4.970322265	4.970322265	2	2	334	334
5	0.47619854	0.47619854	4.523886134	4.523886134	32	32	304	304
5	1.250021169	1.250021169	3.750063506	3.750063506	84	84	252	252
5	2.500042337	2.500042337	2.500042337	2.500042337	168	168	168	168
5	3.750063506	3.750063506	1.250021169	1.250021169	252	252	84	84
5	4.523886134	4.523886134	0.47619854	0.47619854	304	304	32	32
5	4.970322265	4.970322265	0.029762409	0.029762409	334	334	2	2
5	5.000084674	5.000084674	0	0	336	336	0	0
10	0	0	10.00011935	10.00011935	0	0	672	672
10	0.08928678	0.08928678	9.910832567	9.910832567	6	6	666	666
10	0.982154579	0.982154579	9.017964768	9.017964768	66	66	606	606
10	2.500029837	2.500029837	7.50008951	7.50008951	168	168	504	504
10	5.000059673	5.000059673	5.000059673	5.000059673	336	336	336	336
10	7.50008951	7.50008951	2.500029837	2.500029837	504	504	168	168
10	9.017964768	9.017964768	0.982154579	0.982154579	606	606	66	66
10	9.910832567	9.910832567	0.08928678	0.08928678	666	666	6	6
10	10.00011935	10.00011935	0	0	672	672	0	0
50	0	0	49.99859677	49.99859677	0	0	3360	3360
50	0.476177112	0.476177112	49.52241965	49.52241965	32	32	3328	3328
50	4.999859677	4.999859677	44.99873709	44.99873709	336	336	3024	3024
50	12.49964919	12.49964919	37.49894757	37.4691865	840	840	2520	2518
50	24.99929838	24.99929838	24.99929838	24.99929838	1680	1680	1680	1680
50	37.49894757	37.49894757	12.49964919	12.49964919	2520	2520	840	840
50	44.99873709	44.99873709	4.999859677	4.999859677	3024	3024	336	336
50	49.52241965	49.52241965	0.476177112	0.476177112	3328	3328	32	32
50	49.99859677	49.99859677	0	0	3360	3360	0	0
100	0	0	99.99219406	99.96243448	0	0	6720	6718
100	0.982066192	0.982066192	99.01012787	99.01012787	66	66	6654	6654
100	9.999219406	9.999219406	89.99297466	89.99297466	672	672	6048	6048
100	24.99804852	24.99804852	74.99414555	74.99414555	1680	1680	5040	5040
100	49.99609703	49.99609703	49.99609703	49.96633745	3360	3360	3360	3358
100	74.99414555	74.99414555	24.99804852	24.96828893	5040	5040	1680	1678
100	89.99297466	89.99297466	9.999219406	9.999219406	6048	6048	672	672
100	99.01012787	99.01012787	0.982066192	0.982066192	6654	6654	66	66
100	99.99219406	99.99219406	0	0	6720	6720	0	0
1000	0	0	999.0228902	998.9931574	0	0	67200	67198
1000	9.990228902	9.990228902	989.0326613	988.9434628	672	672	66528	66522
1000	99.90228902	99.90228902	899.1206012	899.0314027	6720	6720	60480	60474
1000	249.7557226	249.7557226	749.2671677	749.207702	16800	16800	50400	50396
1000	499.5114451	499.5114451	499.5114451	499.4222466	33600	33600	33600	33594
1000	749.2671677	749.2671677	249.7557226	249.7259897	50400	50400	16800	16798
1000	899.1206012	899.1206012	99.90228902	99.8725562	60480	60480	6720	6718
1000	989.0326613	989.0326613	9.990228902	9.990228902	66528	66528	672	672
1000	999.0228902	999.0228902	0	0	67200	67200	0	0

Table S2. HPV-EM estimated HPV genotypes for 304 TCGA primary cervical cancer samples.

TCGA Case ID	Total number of reads (human and other)	Included in TCGA-CESC consortium study?	TCGA final genotype	TCGA HPV genotypes	HPV-EM estimated HPV genotypes HPV genotype (percentage, number of reads)
TCGA-BI-A0VR	185589496	TRUE	HPV16	HPV16	HPV16(1.00000,13880);
TCGA-BI-A0VS	213513044	TRUE	HPV16	HPV16	HPV16(1.00000,11473);
TCGA-BI-A20A	156561650	TRUE	HPV16	HPV16	HPV16(1.00000,23531);
TCGA-C5-A0TN	116784232	TRUE	HPV16	HPV16	HPV16(1.00000,6302);
TCGA-C5-A1BE	172988926	TRUE	HPV16	HPV16	HPV16(1.00000,6226);
TCGA-C5-A1BF	167779210	TRUE	HPV18	HPV18	HPV18(1.00000,2931);
TCGA-C5-A1BI	224577562	TRUE	HPV16	HPV16	HPV16(1.00000,84303);
TCGA-C5-A1BJ	269369564	TRUE	HPV16	HPV16	HPV16(1.00000,32725);
TCGA-C5-A1BK	164493332	TRUE	HPV16	HPV16	HPV16(1.00000,6670);
TCGA-C5-A1BL	130387170	TRUE	HPV16	HPV16	HPV16(1.00000,12897);
TCGA-C5-A1BM	189022812	TRUE	HPV18	HPV18	HPV18(1.00000,4051);
TCGA-C5-A1BN	154285800	TRUE	HPV45	HPV45	HPV45(1.00000,18535);
TCGA-C5-A1BQ	190791424	TRUE	HPV31	HPV31	HPV31(1.00000,30579);
TCGA-C5-A1M5	180163748	TRUE	HPV33	HPV33	HPV33(1.00000,92726);
TCGA-C5-A1M6	124340630	TRUE	HPV18	HPV18	HPV18(1.00000,6856);
TCGA-C5-A1M7	195128558	TRUE	HPV18	HPV18	HPV18(1.00000,10819);
TCGA-C5-A1M8	195313702	TRUE	HPV16	HPV16	HPV16(1.00000,3270);
TCGA-C5-A1M9	205203686	TRUE	HPV16	HPV16	HPV16(1.00000,78530);
TCGA-C5-A1ME	119763670	TRUE	HPV18	HPV18	HPV18(1.00000,3506);
TCGA-C5-A1MF	146262734	TRUE	HPV45	HPV45	HPV45(1.00000,6166);
TCGA-C5-A1MH	173349244	TRUE	HPV16	HPV16	HPV16(1.00000,14118);
TCGA-C5-A1MI	201152538	TRUE	HPV18	HPV18	HPV18(1.00000,70753);
TCGA-C5-A1MJ	192245014	TRUE	HPV18	HPV18	HPV18(1.00000,52063);
TCGA-C5-A1MK	145861940	TRUE	HPV58	HPV58	HPV58(1.00000,44832);
TCGA-C5-A1ML	100978028	TRUE	HPV16	HPV16	HPV16(1.00000,15341);
TCGA-C5-A1MN	138072458	TRUE	HPV16	HPV16	HPV16(1.00000,17590);
TCGA-C5-A1MP	123941646	TRUE	HPV16	HPV16	HPV16(1.00000,14118);
TCGA-C5-A2LS	127365682	TRUE	HPV16	HPV16	HPV16(1.00000,21913);
TCGA-C5-A2LT	158958028	TRUE	HPV70	HPV70	HPV70(1.00000,9442);
TCGA-C5-A2LV	178111436	TRUE	HPV16	HPV16	HPV16(1.00000,16714);
TCGA-C5-A2LX	177957202	TRUE	HPV16	HPV16	HPV16(1.00000,30408);
TCGA-C5-A2LY	109124246	TRUE	HPV16	HPV16	HPV16(1.00000,18494);
TCGA-C5-A2LZ	158132126	TRUE	HPV16	HPV16	HPV16(1.00000,36590);
TCGA-C5-A2M1	241042170	TRUE	HPV16	HPV16	HPV16(1.00000,24850);
TCGA-C5-A2M2	133945736	TRUE	HPV16	HPV16	HPV16(1.00000,27827);
TCGA-C5-A3HD	152410312	TRUE	HPV16	HPV16	HPV16(1.00000,63805);
TCGA-C5-A3HE	165779472	TRUE	HPV18	HPV18	HPV18(1.00000,9714);
TCGA-C5-A3HF	160128448	TRUE	HPV18	HPV18, HPV58	HPV18(0.86075,30197);HPV58(0.13925,4885);
TCGA-C5-A3HL	169261628	TRUE	HPV16	HPV16	HPV16(1.00000,36951);
TCGA-C5-A7CG	134534760	TRUE	HPV52	HPV52	HPV52(1.00000,32371);
TCGA-C5-A7CH	116906114	TRUE	HPV16	HPV16	HPV16(1.00000,30160);
TCGA-C5-A7CJ	121229332	TRUE	HPV16	HPV16	HPV16(1.00000,12067);
TCGA-C5-A7CK	158440118	TRUE	HPV16	HPV16	HPV16(1.00000,32689);
TCGA-C5-A7CL	131426104	TRUE	HPV16	HPV16	HPV16(1.00000,14556);
TCGA-C5-A7CM	124539504	TRUE	HPV18	HPV18	HPV18(1.00000,3371);
TCGA-C5-A7UE	108673304	TRUE	HPV16	HPV16	HPV16(1.00000,5711);
TCGA-DG-A2KH	190670394	TRUE	HPV18	HPV18	HPV18(1.00000,37049);
TCGA-DG-A2KJ	70557280	TRUE	HPV18	HPV18	HPV18(1.00000,317);
TCGA-DG-A2KK	165519920	TRUE	HPV16	HPV16	HPV16(1.00000,39398);
TCGA-DG-A2KL	171143958	TRUE	HPV16	HPV16	HPV16(1.00000,21931);
TCGA-DG-A2KM	200834926	TRUE	HPV16	HPV16	HPV16(1.00000,4383);
TCGA-DR-A0ZL	166048866	TRUE	HPV16	HPV16	HPV16(1.00000,18773);
TCGA-DR-A0ZM	159222338	TRUE	negative	negative	negative
TCGA-DS-A0VK	198944394	TRUE	HPV16	HPV16	HPV16(1.00000,28904);
TCGA-DS-A0VL	167589344	TRUE	HPV16	HPV16	HPV16(1.00000,32095);
TCGA-DS-A0VM	192550376	TRUE	HPV16	HPV16	HPV16(1.00000,36579);
TCGA-DS-A0VN	211369374	TRUE	HPV16	HPV16	HPV16(1.00000,22660);
TCGA-DS-A3LQ	179331904	TRUE	HPV69	HPV69	HPV69(0.98380,17487);HPV26(0.01620,288);
TCGA-DS-A5RQ	114479366	TRUE	HPV16	HPV16	HPV16(1.00000,8025);
TCGA-EA-A1QS	170857858	TRUE	HPV39	HPV39	HPV39(1.00000,28590);
TCGA-EA-A1QT	220978146	TRUE	HPV16	HPV16	HPV16(1.00000,50523);
TCGA-EA-A3HQ	219433742	TRUE	HPV16	HPV16	HPV16(1.00000,49123);
TCGA-EA-A3HR	199731584	TRUE	HPV73	HPV73	HPV73(1.00000,86357);
TCGA-EA-A3HT	188388036	TRUE	HPV16	HPV16	HPV16(1.00000,28730);
TCGA-EA-A3HU	184450416	TRUE	HPV16	HPV16	HPV16(1.00000,30550);
TCGA-EA-A3QD	151255232	TRUE	HPV16	HPV16	HPV16(1.00000,31454);
TCGA-EA-A3QE	210394088	TRUE	HPV16	HPV16	HPV16(1.00000,18278);

TCGA-EA-A3Y4	183627576	TRUE	HPV45	HPV45	HPV45(1.00000,2214);
TCGA-EA-A410	122689004	TRUE	negative	negative	negative
TCGA-EA-A411	213112768	TRUE	HPV16	HPV16	HPV16(1.00000,30851);
TCGA-EA-A439	223993818	TRUE	HPV18	HPV18	HPV18(1.00000,80659);
TCGA-EA-A43B	94731752	TRUE	HPV16	HPV16	HPV16(1.00000,11019);
TCGA-EA-A44S	134164504	TRUE	HPV16	HPV16	HPV16(1.00000,946);
TCGA-EA-A4BA	113311950	TRUE	negative	negative	negative
TCGA-EA-A50E	119383610	TRUE	HPV16	HPV16	HPV16(1.00000,1504);
TCGA-EA-A556	118533046	TRUE	negative	negative	negative
TCGA-EA-A5FO	106251504	TRUE	HPV16	HPV16	HPV16(1.00000,26415);
TCGA-EA-A5O9	128447396	TRUE	HPV16	HPV16	HPV16(1.00000,9269);
TCGA-EA-A5ZD	117756132	TRUE	HPV58	HPV58	HPV58(1.00000,6126);
TCGA-EA-A5ZE	117664606	TRUE	HPV59	HPV59	HPV59(1.00000,1667);
TCGA-EA-A5ZF	117152630	TRUE	HPV18	HPV18	HPV18(1.00000,9722);
TCGA-EA-A6QX	123972056	TRUE	HPV58	HPV58	HPV58(1.00000,24811);
TCGA-EA-A78R	109146896	TRUE	HPV52	HPV52	HPV52(1.00000,731);
TCGA-EK-A2GZ	193948022	TRUE	HPV52	HPV52	HPV52(1.00000,18784);
TCGA-EK-A2H0	165262444	TRUE	HPV68	HPV68	HPV68(0.88655,25445);HPV39(0.09233,2650);HPV70(0.02111,606);
TCGA-EK-A2H1	140289936	TRUE	HPV16	HPV16	HPV16(1.00000,5327);
TCGA-EK-A2IP	162277028	TRUE	HPV16	HPV16, HPV45	HPV16(0.92145,14640);HPV45(0.07855,1248);
TCGA-EK-A2PG	172483142	TRUE	HPV58	HPV58	HPV58(1.00000,56930);
TCGA-EK-A2PI	172409678	TRUE	HPV16	HPV16	HPV16(1.00000,22956);
TCGA-EK-A2PK	150947066	TRUE	HPV18	HPV18	HPV18(1.00000,6987);
TCGA-EK-A2PL	102293976	TRUE	HPV39	HPV39	HPV39(1.00000,1585);
TCGA-EK-A2PM	149671542	TRUE	negative	negative	negative
TCGA-EK-A2R7	178553622	TRUE	HPV18	HPV18	HPV18(1.00000,7068);
TCGA-EK-A2R8	131041726	TRUE	HPV45	HPV45	HPV45(1.00000,42901);
TCGA-EK-A2R9	157198940	TRUE	HPV33	HPV33	HPV33(1.00000,36754);
TCGA-EK-A2RA	178992420	TRUE	HPV52	HPV52	HPV52(1.00000,7284);
TCGA-EK-A2RB	162665296	TRUE	HPV16	HPV16	HPV16(1.00000,35207);
TCGA-EK-A2RC	145809076	TRUE	HPV16	HPV16	HPV16(1.00000,3648);
TCGA-EK-A2RE	179869516	TRUE	HPV16	HPV16	HPV16(1.00000,9211);
TCGA-EK-A2RJ	170026798	TRUE	HPV59	HPV59	HPV59(1.00000,2412);
TCGA-EK-A2RK	176351472	TRUE	HPV16	HPV16	HPV16(1.00000,4074);
TCGA-EK-A2RL	202624576	TRUE	HPV16	HPV16	HPV16(1.00000,24143);
TCGA-EK-A2RM	132638992	TRUE	HPV18	HPV18	HPV18(1.00000,17782);
TCGA-EK-A2RN	164630764	TRUE	HPV16	HPV16	HPV16(1.00000,8886);
TCGA-EK-A2RO	121208882	TRUE	HPV16	HPV16	HPV16(1.00000,1157);
TCGA-EK-A3GJ	167718684	TRUE	HPV58	HPV58	HPV58(1.00000,15569);
TCGA-EK-A3GK	183379806	TRUE	HPV16	HPV16	HPV16(1.00000,17479);
TCGA-EK-A3GM	186122872	TRUE	HPV45	HPV45	HPV45(1.00000,20630);
TCGA-EK-A3GN	178293658	TRUE	HPV16	HPV16	HPV16(1.00000,39350);
TCGA-EX-A1H5	167756340	TRUE	HPV16	HPV16	HPV16(1.00000,23780);
TCGA-EX-A1H6	173654858	TRUE	HPV16	HPV16	HPV16(1.00000,1511);
TCGA-EX-A3L1	148176098	TRUE	HPV45	HPV45	HPV45(1.00000,14585);
TCGA-EX-A69L	123837620	TRUE	HPV16	HPV16	HPV16(1.00000,27806);
TCGA-EX-A69M	127500764	TRUE	HPV52	HPV52	HPV52(1.00000,21279);
TCGA-FU-A23K	170111686	TRUE	HPV18	HPV18	HPV18(1.00000,19430);
TCGA-FU-A23L	197976316	TRUE	HPV45	HPV45	HPV45(1.00000,37830);
TCGA-FU-A2QG	188482202	TRUE	HPV16	HPV16	HPV16(1.00000,36199);
TCGA-FU-A3EO	125290464	TRUE	HPV16	HPV16	HPV16(1.00000,8618);
TCGA-FU-A3HY	169013538	TRUE	HPV16	HPV16	HPV16(1.00000,53336);
TCGA-FU-A3NI	197807388	TRUE	HPV16	HPV16	HPV16(1.00000,20936);
TCGA-FU-A3TQ	198778004	TRUE	HPV16	HPV16	HPV16(1.00000,21587);
TCGA-FU-A3TX	186706050	TRUE	HPV18	HPV18	HPV18(1.00000,34523);
TCGA-FU-A3WB	182747060	TRUE	HPV16	HPV16	HPV16(1.00000,20655);
TCGA-FU-A3YQ	181876636	TRUE	HPV16	HPV16	HPV16(1.00000,20496);
TCGA-FU-A40J	179750602	TRUE	HPV16	HPV16	HPV16(1.00000,34083);
TCGA-FU-A57G	121822452	TRUE	negative	negative	negative
TCGA-FU-A5XV	121981284	TRUE	HPV18	HPV18	HPV18(1.00000,4044);
TCGA-FU-A770	108346416	TRUE	HPV16	HPV16	HPV16(1.00000,49710);
TCGA-HG-A2PA	186139964	TRUE	HPV16	HPV16	HPV16(1.00000,5847);
TCGA-HM-A3JJ	182720030	TRUE	HPV16	HPV16	HPV16(1.00000,6909);
TCGA-HM-A3JK	115799274	TRUE	HPV35	HPV35	HPV35(1.00000,22513);
TCGA-HM-A4S6	121622128	TRUE	HPV16	HPV16	HPV16(1.00000,10200);
TCGA-HM-A6W2	134397052	TRUE	negative	negative	negative
TCGA-IR-A3L7	164042494	TRUE	HPV18	HPV18	HPV18(1.00000,22647);
TCGA-IR-A3LA	156489958	TRUE	negative	negative	negative
TCGA-IR-A3LB	214474466	TRUE	HPV45	HPV45	HPV45(0.98643,77567);HPV18(0.01357,1067);
TCGA-IR-A3LC	184144666	TRUE	HPV16	HPV16	HPV16(1.00000,26337);
TCGA-IR-A3LF	153745238	TRUE	HPV16	HPV16	HPV16(1.00000,37425);
TCGA-IR-A3LH	183089538	TRUE	HPV45	HPV45	HPV45(1.00000,10934);
TCGA-IR-A3LI	115631380	TRUE	HPV16	HPV16	HPV16(1.00000,5810);
TCGA-IR-A3LK	160627918	TRUE	HPV31	HPV31	HPV31(1.00000,56193);
TCGA-IR-A3LL	170647876	TRUE	HPV16	HPV16	HPV16(1.00000,19782);
TCGA-JW-A5VG	135818436	TRUE	HPV59	HPV59	HPV59(1.00000,12081);
TCGA-JW-A5VH	125276408	TRUE	negative	negative	negative
TCGA-JW-A5VI	129985936	TRUE	HPV30	HPV30	HPV30(1.00000,29488);
TCGA-JW-A5VJ	144224034	TRUE	HPV18	HPV18	HPV18(1.00000,3379);
TCGA-JW-A5VK	130562068	TRUE	HPV16	HPV16, HPV26	HPV26(0.99089,10552);HPV16(0.00911,97);
TCGA-JW-A5VL	135593816	TRUE	HPV16	HPV16	HPV16(1.00000,97375);
TCGA-JW-A69B	169697358	TRUE	HPV16	HPV16	HPV16(1.00000,22937);
TCGA-JX-A3PZ	138677222	TRUE	HPV45	HPV45	HPV45(1.00000,26160);
TCGA-JX-A3Q0	137832850	TRUE	HPV31	HPV31	HPV31(1.00000,20176);

TCGA-JX-A3Q8	149069880	TRUE	HPV16	HPV16	HPV16(1.00000,43723);
TCGA-JX-A5QV	125022170	TRUE	HPV16	HPV16	HPV16(1.00000,23919);
TCGA-LP-A4AU	144145962	TRUE	HPV18	HPV18	HPV18(1.00000,6876);
TCGA-LP-A4AV	90983618	TRUE	HPV56	HPV56	HPV56(1.00000,14618);
TCGA-LP-A4AW	117476674	TRUE	HPV16	HPV16	HPV16(1.00000,2242);
TCGA-LP-A4AX	99635454	TRUE	HPV16	HPV16	HPV16(1.00000,14794);
TCGA-LP-A5U2	128850420	TRUE	HPV16	HPV16	HPV16(1.00000,6222);
TCGA-LP-A5U3	124201242	TRUE	HPV16	HPV16	HPV16(1.00000,22348);
TCGA-LP-A7HU	125535584	TRUE	HPV16	HPV16	HPV16(1.00000,15203);
TCGA-MU-A51Y	127565570	TRUE	HPV16	HPV16	HPV16(1.00000,7492);
TCGA-MU-A5YI	120810588	TRUE	HPV16	HPV16	HPV16(1.00000,2908);
TCGA-MY-A5BD	110735410	TRUE	HPV16	HPV16	HPV16(1.00000,4100);
TCGA-MY-A5BE	128034638	TRUE	HPV16	HPV16	HPV16(1.00000,3472);
TCGA-MY-A5BF	117702814	TRUE	HPV16	HPV16	HPV16(1.00000,11446);
TCGA-Q1-A5R1	108964658	TRUE	HPV16	HPV16	HPV16(1.00000,41569);
TCGA-Q1-A5R2	129275516	TRUE	HPV16	HPV16	HPV16(1.00000,24061);
TCGA-Q1-A5R3	190883788	TRUE	HPV18	HPV18	HPV18(1.00000,4966);
TCGA-Q1-A6DT	109662466	TRUE	HPV16	HPV16	HPV16(1.00000,69717);
TCGA-Q1-A6DV	128187402	TRUE	HPV16	HPV16	HPV16(1.00000,31177);
TCGA-Q1-A6DW	130034222	TRUE	HPV16	HPV16	HPV16(1.00000,55538);
TCGA-Q1-A73O	150333512	TRUE	HPV18	HPV18	HPV18(0.99705,39241);HPV97(0.00295,116);
TCGA-Q1-A73P	122749276	TRUE	HPV18	HPV18	HPV18(1.00000,93076);
TCGA-Q1-A73Q	120950762	TRUE	HPV33	HPV33	HPV33(0.99568,53760);HPV18(0.00432,233);
TCGA-Q1-A73R	182850320	TRUE	HPV16	HPV16	HPV16(1.00000,71880);
TCGA-Q1-A73S	99980890	TRUE	HPV18	HPV18	HPV18(1.00000,7084);
TCGA-R2-A69V	91910852	TRUE	HPV39	HPV39	HPV39(1.00000,3791);
TCGA-RA-A741	107668188	TRUE	HPV16	HPV16	HPV16(1.00000,6755);
TCGA-2W-A8YY	131365902	FALSE	NA	NA	negative
TCGA-4J-AA1J	110121082	FALSE	NA	NA	HPV18(1.00000,66822);
TCGA-C5-A1MQ	121910178	FALSE	NA	NA	HPV18(1.00000,6396);
TCGA-C5-A7CO	135471400	FALSE	NA	NA	HPV31(1.00000,18162);
TCGA-C5-A7UC	126035380	FALSE	NA	NA	HPV45(1.00000,10765);
TCGA-C5-A7UH	130018114	FALSE	NA	NA	HPV16(1.00000,31395);
TCGA-C5-A7UI	120459186	FALSE	NA	NA	HPV33(1.00000,1381);
TCGA-C5-A7X3	112079078	FALSE	NA	NA	HPV45(1.00000,21991);
TCGA-C5-A7X5	142266780	FALSE	NA	NA	HPV35(1.00000,36408);
TCGA-C5-A7X8	115659860	FALSE	NA	NA	HPV18(1.00000,41618);
TCGA-C5-A7XC	105632188	FALSE	NA	NA	HPV16(1.00000,4068);
TCGA-C5-A8XH	151001352	FALSE	NA	NA	HPV16(1.00000,13176);
TCGA-C5-A8XI	142047960	FALSE	NA	NA	HPV52(1.00000,15154);
TCGA-C5-A8XJ	125118432	FALSE	NA	NA	HPV33(1.00000,19857);
TCGA-C5-A8XK	122116342	FALSE	NA	NA	HPV16(1.00000,22775);
TCGA-C5-A8YQ	132578268	FALSE	NA	NA	negative
TCGA-C5-A8YR	110167904	FALSE	NA	NA	HPV45(1.00000,35741);
TCGA-C5-A8YT	132283382	FALSE	NA	NA	negative
TCGA-C5-A8ZZ	132968336	FALSE	NA	NA	HPV16(1.00000,2493);
TCGA-C5-A901	142060616	FALSE	NA	NA	HPV16(1.00000,9575);
TCGA-C5-A902	134057648	FALSE	NA	NA	HPV16(1.00000,25946);
TCGA-C5-A905	138351226	FALSE	NA	NA	HPV16(1.00000,29635);
TCGA-C5-A907	112676244	FALSE	NA	NA	HPV18(1.00000,24724);
TCGA-DS-A1O9	227113924	FALSE	NA	NA	HPV73(1.00000,36240);
TCGA-DS-A1OA	154986580	FALSE	NA	NA	HPV58(1.00000,36844);
TCGA-DS-A1OB	176774446	FALSE	NA	NA	HPV31(1.00000,24094);
TCGA-DS-A1OC	119425746	FALSE	NA	NA	HPV16(1.00000,16905);
TCGA-DS-A1OD	225511600	FALSE	NA	NA	HPV16(1.00000,19812);
TCGA-DS-A7WF	137415934	FALSE	NA	NA	HPV16(1.00000,12731);
TCGA-DS-A7WH	126702404	FALSE	NA	NA	HPV16(1.00000,19082);
TCGA-DS-A7WI	118038208	FALSE	NA	NA	HPV16(1.00000,9809);
TCGA-EA-A3HS	178877514	FALSE	NA	NA	HPV16(1.00000,24871);
TCGA-EA-A97N	101353314	FALSE	NA	NA	HPV16(1.00000,16043);
TCGA-EK-A2IR	193053782	FALSE	NA	NA	HPV16(1.00000,37339);
TCGA-EX-A449	128342370	FALSE	NA	NA	HPV16(1.00000,42738);
TCGA-EX-A8YF	132449126	FALSE	NA	NA	HPV18(1.00000,6582);
TCGA-FU-A3HZ	94204342	FALSE	NA	NA	negative
TCGA-GH-A9DA	135977610	FALSE	NA	NA	HPV45(1.00000,17952);
TCGA-JW-A852	107020614	FALSE	NA	NA	HPV16(1.00000,517);
TCGA-JW-AAVH	105527326	FALSE	NA	NA	HPV16(1.00000,12847);
TCGA-MA-AA3W	102995270	FALSE	NA	NA	HPV16(1.00000,11088);
TCGA-MA-AA3X	116832774	FALSE	NA	NA	HPV16(1.00000,54155);
TCGA-MA-AA3Y	143754708	FALSE	NA	NA	HPV39(1.00000,33934);
TCGA-MA-AA3Z	96642696	FALSE	NA	NA	HPV16(1.00000,6097);
TCGA-MA-AA41	115431634	FALSE	NA	NA	HPV16(1.00000,43259);
TCGA-MA-AA42	107310162	FALSE	NA	NA	HPV16(1.00000,17869);
TCGA-MA-AA43	122571364	FALSE	NA	NA	HPV18(1.00000,4055);
TCGA-MU-A8JM	137607046	FALSE	NA	NA	HPV16(1.00000,33900);
TCGA-MY-A913	142733356	FALSE	NA	NA	HPV45(1.00000,61195);
TCGA-PN-A8MA	112728056	FALSE	NA	NA	HPV16(1.00000,3530);
TCGA-UC-A7PD	140073168	FALSE	NA	NA	HPV16(1.00000,5040);
TCGA-UC-A7PF	114118350	FALSE	NA	NA	HPV16(1.00000,12396);
TCGA-UC-A7PG	134624262	FALSE	NA	NA	HPV16(1.00000,1990);
TCGA-UC-A7PI	135580420	FALSE	NA	NA	HPV18(1.00000,39295);
TCGA-VS-A8EB	129865718	FALSE	NA	NA	HPV16(1.00000,31697);
TCGA-VS-A8EC	121247134	FALSE	NA	NA	HPV16(1.00000,17689);
TCGA-VS-A8EG	171113516	FALSE	NA	NA	HPV16(1.00000,4749);

TCGA-VS-A8EH	135505590	FALSE	NA	NA	HPV16(1.00000,5012);
TCGA-VS-A8EI	122510556	FALSE	NA	NA	HPV16(1.00000,6023);
TCGA-VS-A8EJ	113231446	FALSE	NA	NA	negative
TCGA-VS-A8EK	129584270	FALSE	NA	NA	HPV35(1.00000,28402);
TCGA-VS-A8EL	139424686	FALSE	NA	NA	HPV31(1.00000,24124);
TCGA-VS-A8Q8	112666812	FALSE	NA	NA	HPV16(1.00000,355);
TCGA-VS-A8Q9	126512642	FALSE	NA	NA	HPV51(1.00000,108032);
TCGA-VS-A8QA	120348074	FALSE	NA	NA	HPV16(1.00000,12123);
TCGA-VS-A8QC	138262090	FALSE	NA	NA	HPV16(1.00000,5214);
TCGA-VS-A8QF	127744744	FALSE	NA	NA	HPV16(1.00000,29996);
TCGA-VS-A8QH	117544734	FALSE	NA	NA	negative
TCGA-VS-A8QM	133576342	FALSE	NA	NA	HPV16(1.00000,13221);
TCGA-VS-A94W	135509386	FALSE	NA	NA	HPV16(1.00000,25678);
TCGA-VS-A94X	116455646	FALSE	NA	NA	HPV45(1.00000,8662);
TCGA-VS-A94Y	89619306	FALSE	NA	NA	HPV45(1.00000,4851);
TCGA-VS-A94Z	119824846	FALSE	NA	NA	HPV16(1.00000,9770);
TCGA-VS-A950	101552934	FALSE	NA	NA	HPV35(1.00000,86538);
TCGA-VS-A952	118356982	FALSE	NA	NA	HPV16(1.00000,18965);
TCGA-VS-A953	161618170	FALSE	NA	NA	HPV16(1.00000,37083);
TCGA-VS-A954	115898050	FALSE	NA	NA	HPV35(1.00000,22867);
TCGA-VS-A957	47901864	FALSE	NA	NA	HPV33(1.00000,12623);
TCGA-VS-A958	89063578	FALSE	NA	NA	HPV39(1.00000,1526);
TCGA-VS-A959	82457646	FALSE	NA	NA	HPV31(1.00000,8463);
TCGA-VS-A9U5	68701186	FALSE	NA	NA	HPV16(1.00000,4144);
TCGA-VS-A9U6	141033252	FALSE	NA	NA	HPV33(1.00000,8692);
TCGA-VS-A9U7	185944176	FALSE	NA	NA	HPV16(1.00000,21214);
TCGA-VS-A9UB	79378434	FALSE	NA	NA	HPV45(1.00000,11084);
TCGA-VS-A9UC	120945668	FALSE	NA	NA	HPV33(1.00000,16559);
TCGA-VS-A9UD	60257468	FALSE	NA	NA	HPV16(1.00000,19206);
TCGA-VS-A9UH	103443422	FALSE	NA	NA	HPV45(1.00000,1493);
TCGA-VS-A9UI	75559928	FALSE	NA	NA	HPV52(1.00000,25052);
TCGA-VS-A9UJ	79290766	FALSE	NA	NA	negative
TCGA-VS-A9UL	97155898	FALSE	NA	NA	HPV18(1.00000,23658);
TCGA-VS-A9UM	94688990	FALSE	NA	NA	HPV16(1.00000,2796);
TCGA-VS-A9UO	192695356	FALSE	NA	NA	HPV18(1.00000,15167);
TCGA-VS-A9UP	97015366	FALSE	NA	NA	HPV18(1.00000,24838);
TCGA-VS-A9UQ	107402002	FALSE	NA	NA	HPV16(1.00000,11284);
TCGA-VS-A9UR	98132098	FALSE	NA	NA	HPV16(1.00000,6135);
TCGA-VS-A9UT	87823052	FALSE	NA	NA	negative
TCGA-VS-A9UU	70382098	FALSE	NA	NA	HPV16(1.00000,3614);
TCGA-VS-A9UV	87931656	FALSE	NA	NA	HPV52(1.00000,8334);
TCGA-VS-A9UY	109346226	FALSE	NA	NA	HPV16(1.00000,12451);
TCGA-VS-A9UZ	85327276	FALSE	NA	NA	HPV16(1.00000,13833);
TCGA-VS-A9V0	48797598	FALSE	NA	NA	negative
TCGA-VS-A9V1	74049578	FALSE	NA	NA	HPV45(1.00000,12455);
TCGA-VS-A9V2	94560068	FALSE	NA	NA	HPV16(1.00000,5242);
TCGA-VS-A9V3	78572614	FALSE	NA	NA	HPV58(1.00000,16902);
TCGA-VS-A9V4	61724840	FALSE	NA	NA	negative
TCGA-VS-A9V5	112531168	FALSE	NA	NA	HPV16(1.00000,48068);
TCGA-VS-AA62	109452816	FALSE	NA	NA	HPV45(1.00000,16415);
TCGA-WL-A834	105467198	FALSE	NA	NA	HPV16(1.00000,15916);
TCGA-XS-A8TJ	136219000	FALSE	NA	NA	HPV16(1.00000,6934);
TCGA-ZJ-A8QO	141446406	FALSE	NA	NA	HPV33(1.00000,37967);
TCGA-ZJ-A8QQ	133976616	FALSE	NA	NA	HPV68(0.90489,15318);HPV39(0.07833,1326);HPV70(0.01678,284);
TCGA-ZJ-A8QR	106049716	FALSE	NA	NA	HPV16(1.00000,19052);
TCGA-ZJ-AAX4	82930894	FALSE	NA	NA	HPV16(1.00000,1865);
TCGA-ZJ-AAX8	128494006	FALSE	NA	NA	HPV70(1.00000,18800);
TCGA-ZJ-AAXA	255335268	FALSE	NA	NA	HPV16(1.00000,22407);
TCGA-ZJ-AAXB	53110604	FALSE	NA	NA	HPV18(1.00000,4181);
TCGA-ZJ-AAXD	57587892	FALSE	NA	NA	HPV16(1.00000,10534);
TCGA-ZJ-AAXF	84952644	FALSE	NA	NA	HPV16(1.00000,3082);
TCGA-ZJ-AAXI	102396968	FALSE	NA	NA	HPV16(1.00000,17838);
TCGA-ZJ-AAXJ	94526746	FALSE	NA	NA	HPV16(1.00000,3368);
TCGA-ZJ-AAXN	231572368	FALSE	NA	NA	HPV45(0.98957,74193);HPV18(0.01043,782);
TCGA-ZJ-AAXT	127555260	FALSE	NA	NA	HPV18(1.00000,2027);
TCGA-ZJ-AAXU	190453134	FALSE	NA	NA	HPV16(1.00000,2646);
TCGA-ZJ-AB0H	88497286	FALSE	NA	NA	HPV18(1.00000,18403);
TCGA-ZJ-AB0I	97339866	FALSE	NA	NA	HPV16(1.00000,7532);
TCGA-ZX-AA5X	70545566	FALSE	NA	NA	HPV35(1.00000,16225);

Table S3. HPV-EM estimated HPV genotypes for 67 WU institutional primary cervical cancer samples.

WU Sample ID	Total number of reads (human and other)	DNA tiling array HPV genotypes	HPV-EM estimated HPV genotypes HPV genotype (percentage, number of reads)
700514_703822	39064908	HPV16	HPV16(1.00000,5644);
700514_704012	34693190	HPV18	HPV18(1.00000,1105);
700514_704397	37417373	HPV16	HPV16(1.00000,2238);
700514_704786	37072245	HPV16	HPV16(1.00000,9820);
700514_704806	36803760	negative	negative
700514_704807	37837113	HPV16	HPV16(1.00000,5938);
700514_704821	29664041	HPV16	HPV16(1.00000,2290);
700514_704956	42889715	HPV58	HPV58(1.00000,7801);
700514_705411	29601306	HPV16	HPV16(0.88065,1675);HPV52(0.11935,227);
700514_705973	43251312	HPV52	HPV52(1.00000,3470);
700514_706069	34944413	HPV16	HPV16(1.00000,3916);
700514_706070	36076809	HPV35	HPV35(1.00000,11971);
700514_706432	33416453	HPV18	HPV18(1.00000,16996);
700514_707268	30709892	HPV16	HPV16(1.00000,3139);
700514_707630	30437912	HPV18	HPV18(1.00000,1180);
700514_707755	26919196	HPV16	HPV16(1.00000,8572);
700514_707913	32325652	HPV16	HPV16(0.90983,1796);HPV82(0.09017,178);
700514_710724	37439590	negative	negative
700514_710725	36189072	HPV59	HPV59(1.00000,4558);
700514_710852	24377034	HPV33	HPV33(1.00000,937);
700514_711012	33494983	HPV16	HPV16(1.00000,10915);
700514_711380	36448212	negative	negative
700514_712739	33988511	HPV45	HPV45(1.00000,10451);
700514_713859	33944191	HPV16	HPV16(1.00000,1161);
700514_713956	40678482	HPV59	HPV59(0.99562,17973);HPV16(0.00438,79);
700514_714694	33794182	HPV16	HPV16(1.00000,11345);
700514_714815	36411838	HPV16	HPV16(1.00000,2401);
700514_714960	37931477	HPV16	HPV16(1.00000,10862);
700514_715842	29214818	negative	negative
700514_720059	34295566	negative	negative
700514_720170	36759701	negative	negative
700514_722396	33627069	HPV16	HPV16(1.00000,6797);
700514_724123	61615145	HPV16	HPV16(1.00000,27759);
700514_724788	49470965	HPV16, HPV18	HPV18(0.99641,27237);HPV16(0.00359,98);
700514_725136	38745369	HPV16	HPV16(1.00000,17199);
700514_725170	32310377	HPV16	HPV16(1.00000,5893);
700514_727879	34709865	HPV16	HPV16(1.00000,17086);
700514_728446	31185192	HPV16	HPV16(1.00000,8931);
700514_728723	31598686	HPV16	HPV16(1.00000,3055);
700514_728836	35065158	HPV18	HPV18(1.00000,13877);
700514_731499	35861894	HPV18	HPV18(1.00000,11654);
700514_731503	36291014	HPV16	HPV16(1.00000,7792);
700514_731976	29387785	HPV16	HPV16(1.00000,3309);
700514_731985	31551421	HPV16	HPV16(1.00000,4887);
700514_732759	33083985	HPV16	HPV16(1.00000,485);
700514_732945	39877824	HPV16	negative
700514_732978	29275651	HPV18	HPV18(1.00000,3038);
700514_733024	31296398	HPV16	HPV16(1.00000,623);
700514_733384	30251169	HPV33	HPV33(1.00000,12509);
700514_733495	35174128	HPV33	HPV33(1.00000,962);
700514_733865	35508472	HPV16	HPV16(1.00000,10583);
700514_735045	28019749	HPV52	HPV52(1.00000,4365);
700514_735379	33909113	HPV16	HPV16(1.00000,5879);
700514_736378	42936410	negative	negative
700514_736462	30715931	HPV16	HPV16(1.00000,3688);
700514_738147	29972226	negative	negative
700514_740791	31322906	negative	HPV16(1.00000,50);
700514_740838	33944274	HPV18	HPV18(1.00000,7255);
700514_745280	32489954	HPV56	HPV56(1.00000,8151);
700514_745454	34462730	HPV16	HPV16(1.00000,1880);
700514_746486	29271861	negative	negative
700514_747167	23943345	HPV16	HPV16(1.00000,3011);
700514_754520	28241349	HPV16	HPV16(1.00000,4774);
700514_755115	27450363	HPV16	HPV16(1.00000,2186);
700514_755438	34489377	HPV16	HPV16(1.00000,2858);
700514_755439	29647336	HPV16, HPV66	HPV44(0.69832,8377);HPV16(0.25067,3007);HPV31(0.04668,560);HPV74(0.00433,52);
700514_760832	26481201	HPV16	HPV16(1.00000,11840);

Table S4. HPV-EM estimated HPV genotypes for 499 TCGA primary head and neck cancer samples.

TCGA Case ID	Total number of reads (human and other)	Included in TCGA-HNSC consortium study?	TCGA final HPV status	TCGA MassArray HPV calls	HPV-EM estimated HPV genotypes HPV genotype (percentage, number of reads)
TCGA-BA-4074	197502140	TRUE	Negative	NA	negative
TCGA-BA-4076	116073560	TRUE	Negative	NA	negative
TCGA-BA-4077	114493356	TRUE	Positive	HPV16	HPV16(1.00000,16685);
TCGA-BA-4078	230078502	TRUE	Negative	NA	negative
TCGA-BA-5151	223854226	TRUE	Negative	NA	negative
TCGA-BA-5152	211731896	TRUE	Negative	HPV16	negative
TCGA-BA-5153	108215774	TRUE	Positive	HPV16	HPV16(1.00000,17575);
TCGA-BA-5555	162942444	TRUE	Negative	NA	negative
TCGA-BA-5556	171009798	TRUE	Negative	NA	negative
TCGA-BA-5557	178800186	TRUE	Negative	NA	negative
TCGA-BA-5558	160127844	TRUE	Negative	NA	negative
TCGA-BA-5559	194079298	TRUE	Positive	HPV16	HPV16(1.00000,17013);
TCGA-BA-6868	213199224	TRUE	Negative	NA	negative
TCGA-BA-6869	153037296	TRUE	Negative	NA	negative
TCGA-BA-6870	205904528	TRUE	Negative	NA	negative
TCGA-BA-6871	188679852	TRUE	Negative	NA	negative
TCGA-BA-6872	104382226	TRUE	Negative	NA	negative
TCGA-BA-6873	88621532	TRUE	Negative	NA	negative
TCGA-BA-7269	141712374	TRUE	Negative	NA	negative
TCGA-BB-4217	165501792	TRUE	Negative	NA	negative
TCGA-BB-4223	111125592	TRUE	Positive	HPV16	HPV16(1.00000,12379);
TCGA-BB-4224	138404774	TRUE	Negative	HPV16	negative
TCGA-BB-4225	151996370	TRUE	Positive	HPV33	HPV33(1.00000,81201);
TCGA-BB-4228	138393802	TRUE	Positive	HPV16, HPV33	HPV16(1.00000,22894);
TCGA-CN-4723	162369252	TRUE	Negative	NA	negative
TCGA-CN-4725	196149014	TRUE	Negative	NA	negative
TCGA-CN-4726	124499276	TRUE	Negative	NA	negative
TCGA-CN-4727	112977104	TRUE	Negative	NA	negative
TCGA-CN-4728	140859982	TRUE	Negative	NA	negative
TCGA-CN-4729	153502766	TRUE	Negative	NA	negative
TCGA-CN-4730	151787132	TRUE	Negative	NA	negative
TCGA-CN-4731	138518870	TRUE	Negative	NA	negative
TCGA-CN-4733	144946808	TRUE	Negative	NA	negative
TCGA-CN-4735	140666922	TRUE	Negative	NA	negative
TCGA-CN-4736	136885368	TRUE	Negative	NA	negative
TCGA-CN-4737	130788170	TRUE	Negative	NA	negative
TCGA-CN-4738	157351602	TRUE	Negative	NA	negative
TCGA-CN-4739	171304188	TRUE	Negative	NA	negative
TCGA-CN-4740	164750444	TRUE	Negative	NA	negative
TCGA-CN-4741	175676074	TRUE	Positive	HPV16	HPV16(1.00000,30438);
TCGA-CN-4742	173890262	TRUE	Negative	NA	negative
TCGA-CN-5355	155358942	TRUE	Negative	NA	negative
TCGA-CN-5356	189816538	TRUE	Negative	NA	negative
TCGA-CN-5358	149444596	TRUE	Negative	NA	negative
TCGA-CN-5359	174600196	TRUE	Negative	NA	negative
TCGA-CN-5360	126416842	TRUE	Negative	NA	negative
TCGA-CN-5363	112766738	TRUE	Negative	NA	negative
TCGA-CN-5364	156824298	TRUE	Negative	HPV16	negative
TCGA-CN-5365	168095808	TRUE	Negative	HPV16	negative
TCGA-CN-5366	177927504	TRUE	Negative	HPV16	negative
TCGA-CN-5367	176795458	TRUE	Negative	NA	negative
TCGA-CN-5369	167574564	TRUE	Negative	NA	negative
TCGA-CN-5370	185429400	TRUE	Negative	NA	negative
TCGA-CN-5373	140255774	TRUE	Negative	NA	negative
TCGA-CN-5374	117708918	TRUE	Positive	HPV16	HPV16(1.00000,8161);
TCGA-CN-6010	151648518	TRUE	Negative	NA	negative
TCGA-CN-6011	97847042	TRUE	Negative	NA	negative
TCGA-CN-6012	129158768	TRUE	Negative	NA	negative
TCGA-CN-6013	136747112	TRUE	Negative	NA	negative
TCGA-CN-6016	126350172	TRUE	Negative	NA	negative
TCGA-CN-6017	116985108	TRUE	Negative	NA	negative
TCGA-CN-6018	131195184	TRUE	Negative	NA	negative
TCGA-CN-6019	113411916	TRUE	Negative	NA	negative

TCGA-CN-6020	133914150	TRUE	Negative	NA	negative
TCGA-CN-6021	126655984	TRUE	Negative	NA	negative
TCGA-CN-6022	148267446	TRUE	Negative	NA	negative
TCGA-CN-6023	230242820	TRUE	Negative	NA	negative
TCGA-CN-6024	131032562	TRUE	Negative	NA	negative
TCGA-CN-6988	170766110	TRUE	Negative	NA	negative
TCGA-CN-6989	176663546	TRUE	Negative	NA	negative
TCGA-CN-6992	190672888	TRUE	Negative	NA	negative
TCGA-CN-6994	114710142	TRUE	Negative	NA	negative
TCGA-CN-6995	141667924	TRUE	Negative	NA	negative
TCGA-CN-6997	202405544	TRUE	Negative	NA	negative
TCGA-CN-6998	198987068	TRUE	Negative	NA	negative
TCGA-CQ-5323	93674002	TRUE	Positive	HPV16	HPV16(1.00000,4013);
TCGA-CQ-5324	104496850	TRUE	Negative	NA	negative
TCGA-CQ-5325	81795182	TRUE	Negative	NA	negative
TCGA-CQ-5326	168842262	TRUE	Negative	NA	negative
TCGA-CQ-5329	96521892	TRUE	Negative	NA	negative
TCGA-CQ-5330	97001606	TRUE	Negative	NA	negative
TCGA-CQ-5331	225877870	TRUE	Negative	NA	negative
TCGA-CQ-5332	104841734	TRUE	Negative	NA	negative
TCGA-CQ-5334	113318390	TRUE	Negative	NA	negative
TCGA-CQ-6218	163176770	TRUE	Negative	NA	negative
TCGA-CQ-6220	126901226	TRUE	Negative	NA	negative
TCGA-CQ-6221	147134538	TRUE	Negative	NA	negative
TCGA-CQ-6223	190172498	TRUE	Negative	NA	negative
TCGA-CQ-6224	183384560	TRUE	Negative	NA	negative
TCGA-CQ-6225	170033514	TRUE	Negative	NA	negative
TCGA-CQ-6227	181550242	TRUE	Negative	NA	negative
TCGA-CQ-6228	166301170	TRUE	Negative	NA	negative
TCGA-CQ-6229	181007620	TRUE	Negative	NA	negative
TCGA-CQ-7065	149152936	TRUE	Negative	NA	negative
TCGA-CQ-7068	166701518	TRUE	Negative	NA	negative
TCGA-CR-5243	156728902	TRUE	Positive	HPV16	HPV16(1.00000,24860);
TCGA-CR-5247	119102932	TRUE	Negative	NA	negative
TCGA-CR-5248	117836210	TRUE	Positive	HPV16	HPV16(1.00000,10633);
TCGA-CR-5249	153228066	TRUE	Positive	HPV16	HPV16(1.00000,20803);
TCGA-CR-5250	132800784	TRUE	Positive	HPV16	HPV16(1.00000,17796);
TCGA-CR-6467	226352666	TRUE	Positive	HPV33	HPV33(1.00000,56289);
TCGA-CR-6470	151494206	TRUE	Positive	HPV16	HPV16(1.00000,12639);
TCGA-CR-6471	165675364	TRUE	Positive	HPV33	HPV33(1.00000,134533);
TCGA-CR-6472	117471774	TRUE	Positive	HPV16	HPV16(1.00000,10630);
TCGA-CR-6473	113113550	TRUE	Positive	HPV16	HPV16(1.00000,5352);
TCGA-CR-6474	195729314	TRUE	Negative	HPV16	negative
TCGA-CR-6477	156185080	TRUE	Negative	NA	negative
TCGA-CR-6478	193917808	TRUE	Negative	NA	negative
TCGA-CR-6481	184930018	TRUE	Positive	HPV16	HPV16(1.00000,11483);
TCGA-CR-6482	153489010	TRUE	Positive	HPV16	HPV16(1.00000,10859);
TCGA-CR-6484	141571000	TRUE	Negative	HPV16	negative
TCGA-CR-6487	173152568	TRUE	Positive	HPV16	HPV16(1.00000,10134);
TCGA-CR-6488	171983058	TRUE	Negative	NA	negative
TCGA-CR-6491	151872830	TRUE	Negative	HPV16	negative
TCGA-CR-6492	174127980	TRUE	Negative	NA	negative
TCGA-CR-6493	145234038	TRUE	Negative	HPV16	negative
TCGA-CR-7364	124438702	TRUE	Negative	NA	negative
TCGA-CR-7365	170762612	TRUE	Negative	NA	negative
TCGA-CR-7367	194398332	TRUE	Negative	NA	negative
TCGA-CR-7368	183648808	TRUE	Positive	HPV16	HPV16(1.00000,39717);
TCGA-CR-7369	157321254	TRUE	Positive	HPV16	HPV16(1.00000,11135);
TCGA-CR-7370	166334108	TRUE	Negative	NA	negative
TCGA-CR-7371	152296240	TRUE	Negative	NA	negative
TCGA-CR-7372	141250358	TRUE	Negative	NA	negative
TCGA-CR-7373	157960404	TRUE	Negative	NA	negative
TCGA-CR-7374	135562300	TRUE	Negative	NA	negative
TCGA-CR-7376	176784482	TRUE	Negative	NA	negative
TCGA-CR-7377	163643598	TRUE	Negative	NA	negative
TCGA-CR-7379	152816760	TRUE	Negative	HPV18	negative
TCGA-CR-7380	116919376	TRUE	Negative	NA	negative
TCGA-CR-7382	164897686	TRUE	Negative	NA	negative
TCGA-CR-7383	158323808	TRUE	Negative	NA	negative
TCGA-CR-7385	78123120	TRUE	Positive	HPV16	HPV16(1.00000,19719);
TCGA-CR-7386	117990234	TRUE	Negative	NA	negative
TCGA-CR-7388	103768196	TRUE	Negative	NA	negative
TCGA-CR-7389	157483888	TRUE	Negative	NA	negative

TCGA-CR-7390	149236986	TRUE	Negative	NA	negative
TCGA-CR-7391	153409542	TRUE	Negative	NA	negative
TCGA-CR-7392	127521198	TRUE	Negative	NA	negative
TCGA-CR-7393	100830316	TRUE	Negative	NA	negative
TCGA-CR-7394	104165092	TRUE	Negative	NA	negative
TCGA-CR-7395	85584832	TRUE	Negative	NA	negative
TCGA-CR-7397	119766428	TRUE	Negative	NA	negative
TCGA-CR-7398	136844726	TRUE	Negative	NA	negative
TCGA-CR-7399	176612534	TRUE	Negative	NA	negative
TCGA-CR-7401	141480380	TRUE	Negative	HPV18	negative
TCGA-CR-7402	210932786	TRUE	Negative	HPV18	negative
TCGA-CR-7404	170957424	TRUE	Positive	NA	HPV16(1.00000,30984);
TCGA-CV-5430	100573952	TRUE	Negative	NA	negative
TCGA-CV-5431	127112416	TRUE	Negative	NA	negative
TCGA-CV-5432	88381894	TRUE	Negative	NA	negative
TCGA-CV-5434	144025990	TRUE	Negative	NA	negative
TCGA-CV-5435	121404364	TRUE	Negative	NA	negative
TCGA-CV-5436	123411192	TRUE	Negative	NA	negative
TCGA-CV-5439	139796090	TRUE	Negative	NA	negative
TCGA-CV-5440	109076964	TRUE	Negative	NA	negative
TCGA-CV-5441	125384316	TRUE	Negative	NA	negative
TCGA-CV-5442	122111200	TRUE	Positive	HPV16	HPV16(1.00000,4186);
TCGA-CV-5443	100740272	TRUE	Positive	HPV16	HPV16(1.00000,11828);
TCGA-CV-5444	113152010	TRUE	Negative	NA	negative
TCGA-CV-5966	143784882	TRUE	Negative	NA	negative
TCGA-CV-5970	139193878	TRUE	Negative	NA	negative
TCGA-CV-5971	174274470	TRUE	Positive	HPV16	HPV16(1.00000,3575);
TCGA-CV-5973	123268466	TRUE	Negative	NA	negative
TCGA-CV-5976	162211886	TRUE	Negative	NA	negative
TCGA-CV-5977	127150010	TRUE	Negative	NA	negative
TCGA-CV-5978	89669118	TRUE	Negative	NA	negative
TCGA-CV-5979	130291390	TRUE	Negative	NA	negative
TCGA-CV-6003	151260158	TRUE	Negative	NA	negative
TCGA-CV-6433	120697768	TRUE	Positive	HPV16	HPV16(1.00000,17492);
TCGA-CV-6436	172189564	TRUE	Negative	NA	negative
TCGA-CV-6441	127704392	TRUE	Negative	NA	negative
TCGA-CV-6933	192534778	TRUE	Negative	NA	negative
TCGA-CV-6934	219596860	TRUE	Negative	NA	negative
TCGA-CV-6935	176316678	TRUE	Negative	NA	negative
TCGA-CV-6936	180956716	TRUE	Negative	NA	negative
TCGA-CV-6937	93891498	TRUE	Negative	NA	negative
TCGA-CV-6938	77422450	TRUE	Negative	NA	negative
TCGA-CV-6939	146976912	TRUE	Positive	HPV33	HPV33(1.00000,9480);
TCGA-CV-6940	133668080	TRUE	Negative	NA	negative
TCGA-CV-6941	147854372	TRUE	Negative	HPV33	negative
TCGA-CV-6942	107928912	TRUE	Negative	NA	negative
TCGA-CV-6943	213463504	TRUE	Negative	NA	negative
TCGA-CV-6945	170750322	TRUE	Negative	NA	negative
TCGA-CV-6948	203276050	TRUE	Negative	NA	negative
TCGA-CV-6950	209822024	TRUE	Negative	NA	negative
TCGA-CV-6951	136990576	TRUE	Negative	NA	negative
TCGA-CV-6952	153893674	TRUE	Negative	NA	negative
TCGA-CV-6953	129031410	TRUE	Negative	NA	negative
TCGA-CV-6954	179652580	TRUE	Negative	NA	negative
TCGA-CV-6955	136394078	TRUE	Negative	NA	negative
TCGA-CV-6959	191277710	TRUE	Negative	NA	negative
TCGA-CV-6960	147754832	TRUE	Negative	NA	negative
TCGA-CV-6961	204863052	TRUE	Positive	HPV16	HPV16(1.00000,23968);
TCGA-CV-6962	135510430	TRUE	Negative	NA	negative
TCGA-CV-7089	156826200	TRUE	Negative	NA	negative
TCGA-CV-7090	126028150	TRUE	Negative	NA	negative
TCGA-CV-7091	164809878	TRUE	Negative	NA	negative
TCGA-CV-7095	115701934	TRUE	Negative	NA	negative
TCGA-CV-7097	153604620	TRUE	Negative	HPV33	negative
TCGA-CV-7099	138378542	TRUE	Negative	NA	negative
TCGA-CV-7100	108496580	TRUE	Positive	HPV33	HPV33(1.00000,17244);
TCGA-CV-7101	158622100	TRUE	Negative	HPV33	negative
TCGA-CV-7102	155754696	TRUE	Negative	HPV33	negative
TCGA-CV-7103	164137964	TRUE	Negative	HPV33	negative
TCGA-CV-7104	132789064	TRUE	Negative	HPV33	negative
TCGA-CV-7177	81037406	TRUE	Negative	NA	negative
TCGA-CV-7178	178180118	TRUE	Negative	NA	negative
TCGA-CV-7180	116674870	TRUE	Negative	NA	negative

TCGA-CV-7183	142679626	TRUE	Negative	NA	negative
TCGA-CV-7235	136332844	TRUE	Negative	NA	negative
TCGA-CV-7236	157440848	TRUE	Negative	NA	negative
TCGA-CV-7238	157348394	TRUE	Negative	NA	negative
TCGA-CV-7242	178376734	TRUE	Negative	NA	negative
TCGA-CV-7245	155098794	TRUE	Negative	NA	negative
TCGA-CV-7247	168076844	TRUE	Negative	NA	negative
TCGA-CV-7248	182709708	TRUE	Negative	NA	negative
TCGA-CV-7250	118804988	TRUE	Negative	NA	negative
TCGA-CV-7252	146389948	TRUE	Negative	NA	negative
TCGA-CV-7253	202651640	TRUE	Negative	NA	negative
TCGA-CV-7254	120626968	TRUE	Negative	NA	negative
TCGA-CV-7255	213787930	TRUE	Negative	NA	negative
TCGA-CV-7261	113439374	TRUE	Negative	NA	negative
TCGA-CV-7263	167162804	TRUE	Negative	NA	negative
TCGA-CV-7406	154347540	TRUE	Positive	HPV16	HPV16(1.00000,2957);
TCGA-CV-7407	159419698	TRUE	Negative	NA	negative
TCGA-CV-7410	169317452	TRUE	Negative	NA	negative
TCGA-CV-7411	180864930	TRUE	Negative	NA	negative
TCGA-CV-7413	161930244	TRUE	Negative	NA	negative
TCGA-CV-7414	171840748	TRUE	Negative	NA	negative
TCGA-CV-7415	175397612	TRUE	Negative	NA	negative
TCGA-CV-7416	162596008	TRUE	Negative	NA	negative
TCGA-CV-7418	156737312	TRUE	Negative	NA	negative
TCGA-CV-7421	148510736	TRUE	Negative	NA	negative
TCGA-CV-7422	173277988	TRUE	Negative	NA	negative
TCGA-CV-7423	162482412	TRUE	Negative	NA	negative
TCGA-CV-7424	157060074	TRUE	Negative	NA	negative
TCGA-CV-7427	166817896	TRUE	Negative	NA	negative
TCGA-CV-7429	146374814	TRUE	Negative	NA	negative
TCGA-CV-7430	171236104	TRUE	Negative	NA	negative
TCGA-CV-7432	155262994	TRUE	Negative	NA	negative
TCGA-CV-7433	167495846	TRUE	Negative	NA	negative
TCGA-CV-7434	167429660	TRUE	Negative	NA	negative
TCGA-CV-7435	157692506	TRUE	Negative	NA	negative
TCGA-CV-7437	142105800	TRUE	Negative	NA	negative
TCGA-CV-7438	137482796	TRUE	Negative	NA	negative
TCGA-CV-7440	149437054	TRUE	Negative	NA	negative
TCGA-CX-7082	162128204	TRUE	Negative	NA	negative
TCGA-CX-7085	152758222	TRUE	Negative	NA	negative
TCGA-CX-7086	161249370	TRUE	Negative	NA	negative
TCGA-CX-7219	170656498	TRUE	Negative	NA	negative
TCGA-D6-6515	145667338	TRUE	Negative	NA	negative
TCGA-D6-6516	162988080	TRUE	Negative	NA	negative
TCGA-D6-6517	182681588	TRUE	Negative	NA	negative
TCGA-D6-6823	201585980	TRUE	Negative	NA	negative
TCGA-D6-6824	149933178	TRUE	Negative	NA	negative
TCGA-D6-6825	146305094	TRUE	Negative	NA	negative
TCGA-D6-6826	215167236	TRUE	Negative	NA	negative
TCGA-DQ-5624	189082814	TRUE	Negative	NA	negative
TCGA-DQ-5625	152684756	TRUE	Negative	NA	negative
TCGA-DQ-5629	115750208	TRUE	Negative	HPV16	negative
TCGA-DQ-5630	133875832	TRUE	Negative	NA	negative
TCGA-DQ-5631	222578446	TRUE	Negative	HPV16	negative
TCGA-DQ-7588	142067228	TRUE	Negative	NA	negative
TCGA-DQ-7591	131921588	TRUE	Positive	HPV35	HPV35(1.00000,36207);
TCGA-DQ-7592	190230162	TRUE	Negative	NA	negative
TCGA-F7-7848	142402516	TRUE	Negative	NA	negative
TCGA-H7-7774	180916638	TRUE	Negative	NA	negative
TCGA-HD-7229	140726426	TRUE	Negative	NA	negative
TCGA-HD-7753	185678620	TRUE	Negative	NA	negative
TCGA-HD-7754	184421120	TRUE	Positive	HPV16	HPV16(1.00000,16179);
TCGA-HD-7831	112059932	TRUE	Negative	HPV16	negative
TCGA-HD-7832	159765326	TRUE	Positive	HPV33	HPV33(1.00000,22143);
TCGA-IQ-7630	183388410	TRUE	Negative	NA	negative
TCGA-IQ-7631	144439426	TRUE	Negative	NA	negative
TCGA-IQ-7632	126722420	TRUE	Negative	NA	negative
TCGA-4P-AA8J	102997962	FALSE	NA	NA	negative
TCGA-BA-4075	118860650	FALSE	NA	NA	negative
TCGA-BA-A4IF	121477722	FALSE	NA	NA	negative
TCGA-BA-A4IG	128646132	FALSE	NA	NA	HPV16(1.00000,28053);
TCGA-BA-A4IH	139320858	FALSE	NA	NA	HPV16(1.00000,44777);
TCGA-BA-A4II	128327002	FALSE	NA	NA	negative

TCGA-BA-A6D8	108052374	FALSE	NA	NA	negative
TCGA-BA-A6DA	104436658	FALSE	NA	NA	negative
TCGA-BA-A6DB	52593102	FALSE	NA	NA	negative
TCGA-BA-A6DD	125758484	FALSE	NA	NA	negative
TCGA-BA-A6DE	148873540	FALSE	NA	NA	negative
TCGA-BA-A6DG	132572288	FALSE	NA	NA	negative
TCGA-BA-A6DI	158438586	FALSE	NA	NA	negative
TCGA-BA-A6DJ	141123098	FALSE	NA	NA	negative
TCGA-BA-A6DL	146169484	FALSE	NA	NA	negative
TCGA-BA-A8YP	128008414	FALSE	NA	NA	negative
TCGA-BB-4227	115525928	FALSE	NA	NA	negative
TCGA-BB-8596	85704282	FALSE	NA	NA	negative
TCGA-BB-8601	91304720	FALSE	NA	NA	negative
TCGA-BB-A5HU	141310364	FALSE	NA	NA	negative
TCGA-BB-A5HY	123689194	FALSE	NA	NA	negative
TCGA-BB-A5HZ	114867622	FALSE	NA	NA	negative
TCGA-BB-A6UM	142652242	FALSE	NA	NA	HPV16(1.00000,5392);
TCGA-BB-A6UO	109593804	FALSE	NA	NA	negative
TCGA-C9-A47Z	127272822	FALSE	NA	NA	negative
TCGA-C9-A480	140595802	FALSE	NA	NA	negative
TCGA-CN-4722	169116194	FALSE	NA	NA	negative
TCGA-CN-4734	159403544	FALSE	NA	NA	negative
TCGA-CN-5361	122600966	FALSE	NA	NA	negative
TCGA-CN-6996	167095748	FALSE	NA	NA	negative
TCGA-CN-A497	106591884	FALSE	NA	NA	negative
TCGA-CN-A498	130176864	FALSE	NA	NA	negative
TCGA-CN-A499	126455852	FALSE	NA	NA	HPV16(1.00000,48568);
TCGA-CN-A49A	126163264	FALSE	NA	NA	negative
TCGA-CN-A49B	122587490	FALSE	NA	NA	negative
TCGA-CN-A49C	115834154	FALSE	NA	NA	HPV16(1.00000,7979);
TCGA-CN-A63T	123034116	FALSE	NA	NA	negative
TCGA-CN-A63U	130267040	FALSE	NA	NA	negative
TCGA-CN-A63V	117388566	FALSE	NA	NA	negative
TCGA-CN-A63W	146058236	FALSE	NA	NA	negative
TCGA-CN-A641	147151950	FALSE	NA	NA	negative
TCGA-CN-A642	124644484	FALSE	NA	NA	negative
TCGA-CN-A6UY	109538498	FALSE	NA	NA	HPV16(1.00000,10873);
TCGA-CN-A6V3	158815708	FALSE	NA	NA	negative
TCGA-CN-A6V6	100127532	FALSE	NA	NA	HPV16(1.00000,10123);
TCGA-CN-A6V7	157413440	FALSE	NA	NA	HPV16(1.00000,8526);
TCGA-CQ-5327	130499634	FALSE	NA	NA	negative
TCGA-CQ-5333	99629568	FALSE	NA	NA	negative
TCGA-CQ-6219	145223104	FALSE	NA	NA	negative
TCGA-CQ-6222	146446566	FALSE	NA	NA	negative
TCGA-CQ-7063	103452130	FALSE	NA	NA	negative
TCGA-CQ-7069	93021130	FALSE	NA	NA	negative
TCGA-CQ-7071	126744414	FALSE	NA	NA	negative
TCGA-CQ-7072	138472494	FALSE	NA	NA	negative
TCGA-CQ-A4C6	138785284	FALSE	NA	NA	negative
TCGA-CQ-A4C7	114725194	FALSE	NA	NA	negative
TCGA-CQ-A4C9	103363822	FALSE	NA	NA	negative
TCGA-CQ-A4CA	111128564	FALSE	NA	NA	negative
TCGA-CQ-A4CB	115451774	FALSE	NA	NA	negative
TCGA-CQ-A4CD	114523458	FALSE	NA	NA	negative
TCGA-CQ-A4CE	131777490	FALSE	NA	NA	negative
TCGA-CQ-A4CG	139332828	FALSE	NA	NA	negative
TCGA-CQ-A4CH	131075020	FALSE	NA	NA	negative
TCGA-CQ-A4CI	125051842	FALSE	NA	NA	negative
TCGA-CR-6480	150592886	FALSE	NA	NA	HPV16(1.00000,21653);
TCGA-CV-7425	144722178	FALSE	NA	NA	negative
TCGA-CV-7428	151382116	FALSE	NA	NA	negative
TCGA-CV-7446	153713924	FALSE	NA	NA	negative
TCGA-CV-7568	194996248	FALSE	NA	NA	negative
TCGA-CV-A45O	122615746	FALSE	NA	NA	negative
TCGA-CV-A45P	135190132	FALSE	NA	NA	negative
TCGA-CV-A45Q	119131094	FALSE	NA	NA	negative
TCGA-CV-A45R	125851454	FALSE	NA	NA	negative
TCGA-CV-A45T	131928502	FALSE	NA	NA	negative
TCGA-CV-A45U	120300062	FALSE	NA	NA	negative
TCGA-CV-A45V	117197904	FALSE	NA	NA	negative
TCGA-CV-A45W	115024592	FALSE	NA	NA	negative
TCGA-CV-A45X	119277236	FALSE	NA	NA	negative
TCGA-CV-A45Y	118140226	FALSE	NA	NA	negative

TCGA-CV-A45Z	116744234	FALSE	NA	NA	negative
TCGA-CV-A460	120324672	FALSE	NA	NA	negative
TCGA-CV-A461	128146814	FALSE	NA	NA	negative
TCGA-CV-A463	123177114	FALSE	NA	NA	negative
TCGA-CV-A464	125502916	FALSE	NA	NA	negative
TCGA-CV-A465	137630306	FALSE	NA	NA	negative
TCGA-CV-A468	122410402	FALSE	NA	NA	negative
TCGA-CV-A6JD	132055198	FALSE	NA	NA	negative
TCGA-CV-A6JE	126867354	FALSE	NA	NA	negative
TCGA-CV-A6JM	129830392	FALSE	NA	NA	negative
TCGA-CV-A6JN	130395828	FALSE	NA	NA	negative
TCGA-CV-A6JO	126013646	FALSE	NA	NA	negative
TCGA-CV-A6JT	124207048	FALSE	NA	NA	negative
TCGA-CV-A6JU	132494310	FALSE	NA	NA	negative
TCGA-CV-A6JY	109842852	FALSE	NA	NA	negative
TCGA-CV-A6JZ	174105910	FALSE	NA	NA	negative
TCGA-CV-A6K0	111613604	FALSE	NA	NA	negative
TCGA-CV-A6K1	136709044	FALSE	NA	NA	negative
TCGA-CV-A6K2	101622374	FALSE	NA	NA	negative
TCGA-CX-A4AQ	118248944	FALSE	NA	NA	negative
TCGA-D6-6827	156190384	FALSE	NA	NA	negative
TCGA-D6-8568	88657008	FALSE	NA	NA	negative
TCGA-D6-8569	85882746	FALSE	NA	NA	negative
TCGA-D6-A4Z9	124616466	FALSE	NA	NA	negative
TCGA-D6-A4ZB	104111750	FALSE	NA	NA	negative
TCGA-D6-A6EK	106976050	FALSE	NA	NA	negative
TCGA-D6-A6EM	109243658	FALSE	NA	NA	negative
TCGA-D6-A6EN	108177198	FALSE	NA	NA	negative
TCGA-D6-A6EO	162905534	FALSE	NA	NA	negative
TCGA-D6-A6EP	121495012	FALSE	NA	NA	negative
TCGA-D6-A6EQ	101150786	FALSE	NA	NA	negative
TCGA-D6-A6ES	125522154	FALSE	NA	NA	negative
TCGA-D6-A74Q	142069958	FALSE	NA	NA	negative
TCGA-F7-8298	86323864	FALSE	NA	NA	negative
TCGA-F7-8489	102379280	FALSE	NA	NA	negative
TCGA-F7-A50G	130161856	FALSE	NA	NA	negative
TCGA-F7-A50I	89845698	FALSE	NA	NA	negative
TCGA-F7-A50J	106026602	FALSE	NA	NA	negative
TCGA-F7-A61S	92313304	FALSE	NA	NA	negative
TCGA-F7-A61V	114255716	FALSE	NA	NA	negative
TCGA-F7-A61W	110084314	FALSE	NA	NA	negative
TCGA-F7-A620	126803252	FALSE	NA	NA	negative
TCGA-F7-A622	120010036	FALSE	NA	NA	negative
TCGA-F7-A623	132152986	FALSE	NA	NA	negative
TCGA-F7-A624	156983562	FALSE	NA	NA	negative
TCGA-H7-8501	87293496	FALSE	NA	NA	negative
TCGA-H7-8502	90278084	FALSE	NA	NA	negative
TCGA-H7-A6C4	139968082	FALSE	NA	NA	negative
TCGA-H7-A76A	108539254	FALSE	NA	NA	HPV16(1.00000,10969);
TCGA-HD-8224	86914220	FALSE	NA	NA	negative
TCGA-HD-8314	95967274	FALSE	NA	NA	HPV35(1.00000,4225);
TCGA-HD-8634	112152076	FALSE	NA	NA	negative
TCGA-HD-8635	97788982	FALSE	NA	NA	negative
TCGA-HD-A4C1	129968340	FALSE	NA	NA	negative
TCGA-HD-A633	147101298	FALSE	NA	NA	negative
TCGA-HD-A634	109989972	FALSE	NA	NA	HPV16(1.00000,59405);
TCGA-HD-A6HZ	99557390	FALSE	NA	NA	negative
TCGA-HD-A6I0	104463462	FALSE	NA	NA	negative
TCGA-HL-7533	124942276	FALSE	NA	NA	HPV16(1.00000,8899);
TCGA-IQ-A61E	121995284	FALSE	NA	NA	negative
TCGA-IQ-A61G	133057126	FALSE	NA	NA	negative
TCGA-IQ-A61H	115620438	FALSE	NA	NA	negative
TCGA-IQ-A61I	134999558	FALSE	NA	NA	HPV35(1.00000,39569);
TCGA-IQ-A61J	145456578	FALSE	NA	NA	negative
TCGA-IQ-A61O	135634528	FALSE	NA	NA	negative
TCGA-IQ-A6SG	163078076	FALSE	NA	NA	negative
TCGA-IQ-A6SH	122769940	FALSE	NA	NA	negative
TCGA-KU-A66S	124385462	FALSE	NA	NA	negative
TCGA-KU-A66T	148145894	FALSE	NA	NA	negative
TCGA-KU-A6H7	138395470	FALSE	NA	NA	HPV16(1.00000,10251);
TCGA-KU-A6H8	120195064	FALSE	NA	NA	negative
TCGA-MT-A51W	132439152	FALSE	NA	NA	negative
TCGA-MT-A51X	124810304	FALSE	NA	NA	negative

TCGA-MT-A67A	85197344	FALSE	NA	NA	negative
TCGA-MT-A67D	135054866	FALSE	NA	NA	negative
TCGA-MT-A67F	117048836	FALSE	NA	NA	negative
TCGA-MT-A7BN	83626422	FALSE	NA	NA	negative
TCGA-MZ-A5BI	121000250	FALSE	NA	NA	HPV33(1.00000,14545);
TCGA-MZ-A6I9	128417624	FALSE	NA	NA	HPV16(1.00000,30638);
TCGA-MZ-A7D7	111659692	FALSE	NA	NA	negative
TCGA-P3-A5Q5	133349128	FALSE	NA	NA	HPV16(1.00000,8352);
TCGA-P3-A5Q6	130412674	FALSE	NA	NA	negative
TCGA-P3-A5QA	135981142	FALSE	NA	NA	negative
TCGA-P3-A5QE	132253980	FALSE	NA	NA	HPV16(1.00000,38401);
TCGA-P3-A5QF	120340302	FALSE	NA	NA	HPV16(1.00000,14106);
TCGA-P3-A6SW	102940504	FALSE	NA	NA	HPV16(1.00000,2372);
TCGA-P3-A6SX	152807474	FALSE	NA	NA	negative
TCGA-P3-A6T0	168455908	FALSE	NA	NA	negative
TCGA-P3-A6T2	136582106	FALSE	NA	NA	negative
TCGA-P3-A6T3	116649448	FALSE	NA	NA	negative
TCGA-P3-A6T4	116629234	FALSE	NA	NA	negative
TCGA-P3-A6T5	147763676	FALSE	NA	NA	negative
TCGA-P3-A6T6	77455002	FALSE	NA	NA	HPV16(1.00000,11039);
TCGA-P3-A6T7	143995114	FALSE	NA	NA	negative
TCGA-P3-A6T8	120154638	FALSE	NA	NA	negative
TCGA-QK-A64Z	138715102	FALSE	NA	NA	negative
TCGA-QK-A652	145806150	FALSE	NA	NA	negative
TCGA-QK-A6IF	83324912	FALSE	NA	NA	HPV16(1.00000,6562);
TCGA-QK-A6IG	96552366	FALSE	NA	NA	negative
TCGA-QK-A6IH	120916260	FALSE	NA	NA	negative
TCGA-QK-A6II	144594692	FALSE	NA	NA	negative
TCGA-QK-A6IJ	126457448	FALSE	NA	NA	negative
TCGA-QK-A6V9	138111476	FALSE	NA	NA	HPV16(1.00000,27485);
TCGA-QK-A6VB	166521350	FALSE	NA	NA	negative
TCGA-QK-A6VC	122736950	FALSE	NA	NA	negative
TCGA-QK-A8Z7	127807548	FALSE	NA	NA	negative
TCGA-QK-A8Z8	126788600	FALSE	NA	NA	negative
TCGA-QK-A8Z9	125524292	FALSE	NA	NA	negative
TCGA-QK-A8ZA	116874478	FALSE	NA	NA	negative
TCGA-QK-A8ZB	195396524	FALSE	NA	NA	negative
TCGA-QK-AA3J	153301758	FALSE	NA	NA	negative
TCGA-QK-AA3K	87219758	FALSE	NA	NA	negative
TCGA-RS-A6TO	89518276	FALSE	NA	NA	negative
TCGA-RS-A6TP	165234468	FALSE	NA	NA	HPV16(1.00000,21404);
TCGA-T2-A6WX	116892190	FALSE	NA	NA	negative
TCGA-T2-A6WZ	77452664	FALSE	NA	NA	negative
TCGA-T2-A6X0	168324838	FALSE	NA	NA	HPV16(1.00000,11114);
TCGA-T2-A6X2	83783670	FALSE	NA	NA	negative
TCGA-T3-A92M	122596654	FALSE	NA	NA	negative
TCGA-T3-A92N	104521002	FALSE	NA	NA	negative
TCGA-TN-A7HI	97596770	FALSE	NA	NA	HPV33(1.00000,14267);
TCGA-TN-A7HJ	117801664	FALSE	NA	NA	negative
TCGA-TN-A7HL	142000430	FALSE	NA	NA	HPV16(1.00000,7049);
TCGA-UF-A718	151436298	FALSE	NA	NA	negative
TCGA-UF-A719	153803482	FALSE	NA	NA	negative
TCGA-UF-A71A	123420664	FALSE	NA	NA	negative
TCGA-UF-A71B	106104728	FALSE	NA	NA	negative
TCGA-UF-A71D	70447760	FALSE	NA	NA	negative
TCGA-UF-A71E	130204320	FALSE	NA	NA	negative
TCGA-UF-A7J9	147928260	FALSE	NA	NA	negative
TCGA-UF-A7JA	83752132	FALSE	NA	NA	negative
TCGA-UF-A7JC	92264040	FALSE	NA	NA	negative
TCGA-UF-A7JD	83439886	FALSE	NA	NA	negative
TCGA-UF-A7JF	106069180	FALSE	NA	NA	negative
TCGA-UF-A7JH	137880040	FALSE	NA	NA	negative
TCGA-UF-A7JJ	92753702	FALSE	NA	NA	negative
TCGA-UF-A7JK	108821700	FALSE	NA	NA	negative
TCGA-UF-A7JO	146722068	FALSE	NA	NA	negative
TCGA-UF-A7JS	91303682	FALSE	NA	NA	negative
TCGA-UF-A7JT	148657832	FALSE	NA	NA	negative
TCGA-UF-A7JV	166792092	FALSE	NA	NA	negative
TCGA-UP-A6WW	168364326	FALSE	NA	NA	HPV16(1.00000,13182);
TCGA-WA-A7GZ	137157004	FALSE	NA	NA	negative
TCGA-WA-A7H4	73105924	FALSE	NA	NA	negative
